# Discovery and quality analysis of a comprehensive set of structural variants and short tandem repeats

**DOI:** 10.1038/s41467-020-16481-5

**Published:** 2020-06-10

**Authors:** David Jakubosky, Erin N. Smith, Matteo D’Antonio, Marc Jan Bonder, William W. Young Greenwald, Agnieszka D’Antonio-Chronowska, Hiroko Matsui, i2QTL Consortium, Marc J. Bonder, Na Cai, Ivan Carcamo-Orive, Matteo D’Antonio, Kelly A. Frazer, William W. Young Greenwald, David Jakubosky, Joshua W. Knowles, Hiroko Matsui, Davis J. McCarthy, Bogdan A. Mirauta, Stephen B. Montgomery, Thomas Quertermous, Daniel D. Seaton, Craig Smail, Erin N. Smith, Oliver Stegle, Oliver Stegle, Stephen B. Montgomery, Christopher DeBoever, Kelly A. Frazer

**Affiliations:** 1 Biomedical Sciences Graduate Program, University of California San Diego, La Jolla, CA 92093-0419 USA; 2 Department of Biomedical Informatics, University of California San Diego, La Jolla, CA 92093-0419 USA; 3 Department of Pediatrics, University of California San Diego, La Jolla, CA 92093 USA; 4 Institute of Genomic Medicine, University of California San Diego, 9500 Gilman Dr, La Jolla, CA 92093 USA; 5 European Molecular Biology Laboratory, European Bioinformatics Institute, Hinxton, Cambridge, UK; 6 European Molecular Biology Laboratory, Genome Biology Unit, Heidelberg, Germany; 7 Bioinformatics and Systems Biology Graduate Program, University of California San Diego, La Jolla, CA USA; 8 Division of Computational Genomics and Systems Genetics, German Cancer Research Center, Heidelberg, Germany; 9 Department of Pathology, Stanford University, Stanford, CA 94305 USA; 10 Department of Genetics, Stanford University, Stanford, CA 94305 USA; 11 Wellcome Sanger Institute, Wellcome Trust Genome Campus, Cambridge, UK; 12 Division of Cardiovascular Medicine and Cardiovascular Institute, Stanford University School of Medicine, Stanford, CA 94305 USA; 13 St Vincent’s Institute of Medical Research, Fitzroy, VIC 3065 Australia; 14 Department of Biomedical Data Science, Stanford University School of Medicine, Stanford, CA 94305 USA

**Keywords:** Bioinformatics, Genotyping and haplotyping, DNA sequencing, Next-generation sequencing, Data processing

## Abstract

Structural variants (SVs) and short tandem repeats (STRs) are important sources of genetic diversity but are not routinely analyzed in genetic studies because they are difficult to accurately identify and genotype. Because SVs and STRs range in size and type, it is necessary to apply multiple algorithms that incorporate different types of evidence from sequencing data and employ complex filtering strategies to discover a comprehensive set of high-quality and reproducible variants. Here we assemble a set of 719 deep whole genome sequencing (WGS) samples (mean 42×) from 477 distinct individuals which we use to discover and genotype a wide spectrum of SV and STR variants using five algorithms. We use 177 unique pairs of genetic replicates to identify factors that affect variant call reproducibility and develop a systematic filtering strategy to create of one of the most complete and well characterized maps of SVs and STRs to date.

## Abstract (web summary)

The complexity of structural variation (SV) and short tandem repeats (STRs) makes it necessary to apply different calling and filtering strategies to sequencing datasets. Here, Jakubosky et al. report a comprehensive SV and STR callset from whole-genome sequencing of 477 individuals from iPSCORE and HipSci using five algorithms.

## Introduction

Structural variants (SVs) and short tandem repeats (STRs) respresent a significant fraction of polymorphic bases in the human genome and have been shown to cause monogenic diseases and contribute to complex disease risk^1–14^. STRs are polymorphic 1–6 base pair (bp) sequence repeats whose total size can range from ~10 bp to more than 1 kb while SVs capture diverse sequence variation greater than 50 bp in size such as insertions, duplications, deletions, and mobile element insertions (MEIs). The full contribution of STRs and SVs to disease risk, quantitative molecular traits, and other human phenotypes is currently not understood because previous studies have typically genotyped SVs and STRs using arrays or low coverage sequencing which are limited in their ability to accurately identify and genotype these variants in many samples across different variant classes and sizes^15–18^. The increasing adoption of high-coverage whole-genome sequencing (WGS) data, however, has recently enabled the development of improved methods to identify STRs and different classes of SVs^19–21^.

While high-depth WGS data have made it possible to profile a wider spectrum of genetic variation, the variability in the size and characteristics of SV classes necessitates the use of several algorithmic approaches that differ in the types of evidence used to capture all classes of SVs. For instance, some algorithms specialize in identifying small SVs (50–5000 bp) by using split or discordant read (abnormal insert size) information to determine the location of SV breakpoints with high resolution^22–25^. Other algorithms detect large SVs (>5 kb) by comparing the amount of reads that align to the reference genome to identify regions that differ in copy number between samples^26–29^, but with lower resolution breakpoint precision^20,30–32^. Finally, algorithms have also been designed to contend with more complex multiallelic signatures, including regions with multiple copy number or repeat alleleles that are more challenging to genotype than biallelic variants^27,29^. Genotyping SVs and STRs across many samples thus requires using several highly parameterized algorithms to discover each class of SVs, processing schemes to combine results from different algorithms, and detailed filtering to remove false positives or inconsistely genotyped variants. Such pipelines for SV/STR identification must also be sensitive to study-specific parameters such as library prepration methods, sequencing depth, cell/tissue type, and read length^19–21,30–32^. Thus, due to the diversity of SV/STR calling algorithms and the need for complex downstream processing, it remains difficult to create a comprehensive SV and STR call set with consistent quality that covers the spectrum of variant sizes and subclasses.

In addition to difficulties associated with complex pipelines for calling SVs and STRs, the need to perform de novo discovery and subsequent genotyping of variants across hundreds or thousands of samples leads to inconsistencies between variant calls across studies. A comprehensive catalog of SVs and STRs in the human genome would make it possible for different studies to genotype this same set of variants. While several efforts are underway to establish such catalogs of SVs^18–20,32–36^ and STRs^37,38^, most are limited in their number and diversity of samples or do not capture all types of variants due to the sequencing depth or algorithms employed. There is also a need to understand the extent to which differences in sample collection and preparation may impact SV and STR calling by measuring the reproducibility of variants called on genetic duplicate samples that share the same genome but were collected and prepped separately. A comprehensive reference catalog of high confidence SVs and STRs discovered in a large set of subjects with deep WGS data could therefore be useful for calling and genotyping the full spectrum of variants across future studies involving hundreds to thousands of subjects.

In this study, as part of the i2QTL consortium, we profile 719 whole genomes from iPSCORE^39–41^ and HipSci^42,43^ with five variant calling algorithms to capture a wide spectrum of SVs including biallelic deletions and duplications; multiallelic copy number variants (mCNVs; regions that have more than two copy number alleles segregating in the population); MEIs; reference MEIs (rMEIs); inversions; unspecified breakends (BND); and STRs. We identify algorithm-specific quality metrics and SV genomic properties associated with the reproducibility of variant calling using 177 pairs of genetic replicates embedded in our collection (25 monozygotic twin pairs and 152 fibroblast–iPSC pairs) and devise filtering and processing approaches to obtain a highly accurate, non-redundant call set across variant classes and algorithmic approaches. We compare our set of SVs with those identified in GTEx^19^ and the 1000 Genomes Project (1KGP)^18^ and find that we capture the vast majority of common SVs likely discoverable in Europeans with short read sequencing and add novel, high-quality variants at lower allele frequencies. Finally, we characterize the extent to which different classes of SVs and STRs are tagged by single-nucleotide polymorphisms (SNPs) and insertions/deletions (indels). This study establishes methods for filtering SVs and STRs to obtain reproducible variant calls and provides a high-quality reference catalog of SVs and STRs that will benefit studies that investigate how these variants contribute to human disease.

## Results

### The i2QTL sample set

**Fig. 1:**
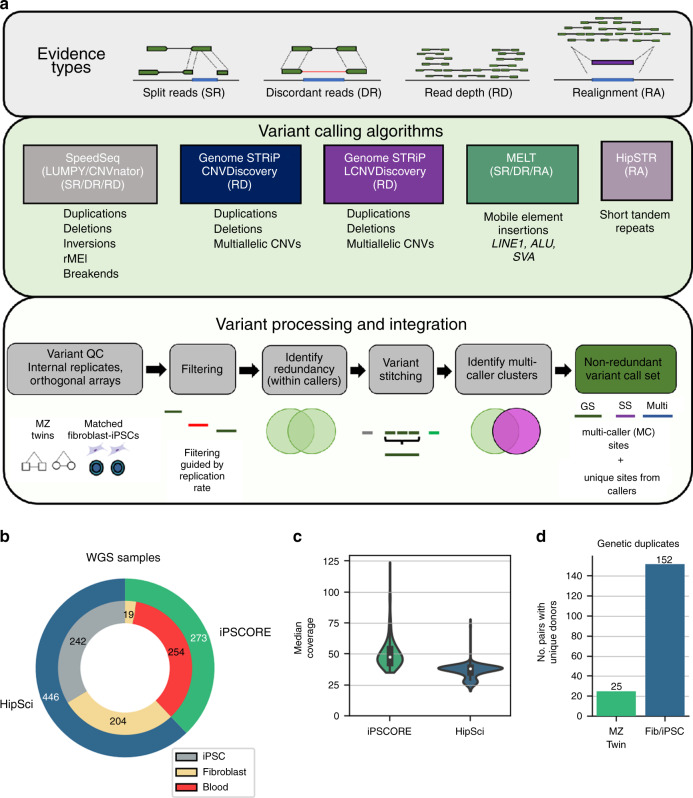
Variant calling, processing, and i2QTL WGS samples. **a** Illustration of the evidence types from short read sequencing data utilized in variant calling (top). Description of the variant callers utilized, the types of variants they identify, and the evidence they use (middle). Flowchart showing the processing, quality control (see Methods), and integration of SVs from different variant callers (bottom). **b** Pie chart showing the number of whole-genome sequencing samples from the iPSCORE or HipSci studies used for variant calling and the cell type from which DNA was obtained. **c** Distribution of the median coverage of whole genomes from iPSCORE (*n* = 273) (green) and HipSci (*n* = 446) (blue). Boxplots are contained within violinplots, and the minimum box edge indicates the first quartile while the maximum box edge indicates the third quartile. White dots in the boxes indicate the median value. Whiskers of the box plot are drawn at the maximum point (upper whisker) or minimum point (lower whisker) that is within 1.5 times the interquartile range (quartile three–quartile one). Points beyond this range are considered outliers (and not plotted) but maximum and minimum values are shown with the range of the outer violinplot. **d** Number of genetic replicate samples included in the collection, including 25 monozygotic twin pairs (iPSCORE) and fibroblast–iPSC pairs from 152 unique donors (HipSci). These data enable robust variant calling for all classes of genetic variation along with reproducibility analysis.

We generated the i2QTL variant calls dataset by calling single-nucleotide variants (SNVs), indels, SVs, and STRs using 719 human WGS samples from 477 unique donors (Fig. 1a, Supplementary Data 1, Supplementary Data 2). The samples were obtained by combining data from two induced pluripoitent stem cell (iPSC) resources: (1) iPSCORE (273 individuals, mean WGS coverage 50×, range 36–126×)^39–41^ and (2) HipSci (446 samples from 204 individuals, mean WGS coverage 37×, range 35–78×)^43,44^ (Fig. 1b, c). The 477 individuals include members of all five 1KGP superpopulations^45^: 415 European, 34 East Asian, 15 Admixed American, 7 South Asian, and 6 African. While all 204 HipSci donors were unrelated, there were 183 donors in iPSCORE that are part of 56 unique families (2–14 individuals/family) (Supplementary Fig. 1), including 25 monozygotic (MZ) twin pairs (Fig. 1d). For 152 HipSci individuals, we also obtained matched fibroblast and iPSC WGS data (Fig. 1d). Between these 152 matched samples and 25 MZ twin pairs, we had WGS data for 177 genetic replicates, which we used to determine quality filtering thresholds and to calculate reproducibility of calls across all variant classes.

### Comprehensive SV call set

To identify SVs across a wide range of sizes (50 bp to >1 Mb) and classes, we called variants using four algorithms (Fig. 1a): SpeedSeq^24,26,46^, Genome STRiP CNVDiscovery, Genome STRiP LCNVDiscovery^29^, and MELT^47^. Together, these algorithms incorporate information from two evidence types: (1) read-pair signal (LUMPY and MELT), which includes detection of split reads (two portions of the same read map to different genomic locations) and discordant read pairs (aligned to the genome with abnormal insert size or orientation) and (2) read-depth (Genome STRiP CNVDiscovery, Genome STRiP LCNVDiscovery, CNVnator). Generally, read-pair signal enables discovery of shorter variants (50 bp) and balanced events, while read-depth signal is limited to discovery of longer (>1 kb) copy number variants (CNVs) which include biallelic deletions, biallelic duplications, and mCNVs. When variant calling algorithms utilize information from a group of samples to predict genotypes, study-specific differences in the WGS data (cell type assayed, library preparation technique) can cause erroneous variant calls. To account for this, we performed variant calling and genotyping separately in HipSci and iPSCORE samples for Genome STRiP and combined variant calls afterward to avoid batch effects during variant calling (Methods). Using read-pair signals we detected 223,371 SVs consisting of CNVs, inversions, MEIs, and novel adjacencies of indeterminate type referred to as BND. Among these SVs, biallelic deletions and biallelic duplications were also supported by supplementary read-depth evidence (CNVnator). Using read-depth signals alone (Genome STRiP), we detected 28,417 biallelic deletions, biallelic duplications, and mCNVs, bringing the initial call set to a combined 251,788 SVs, before additional processing (Supplementary Figs. 2–6).

### Reproducibility of SV calling is associated with quality metrics

**Fig. 2:**
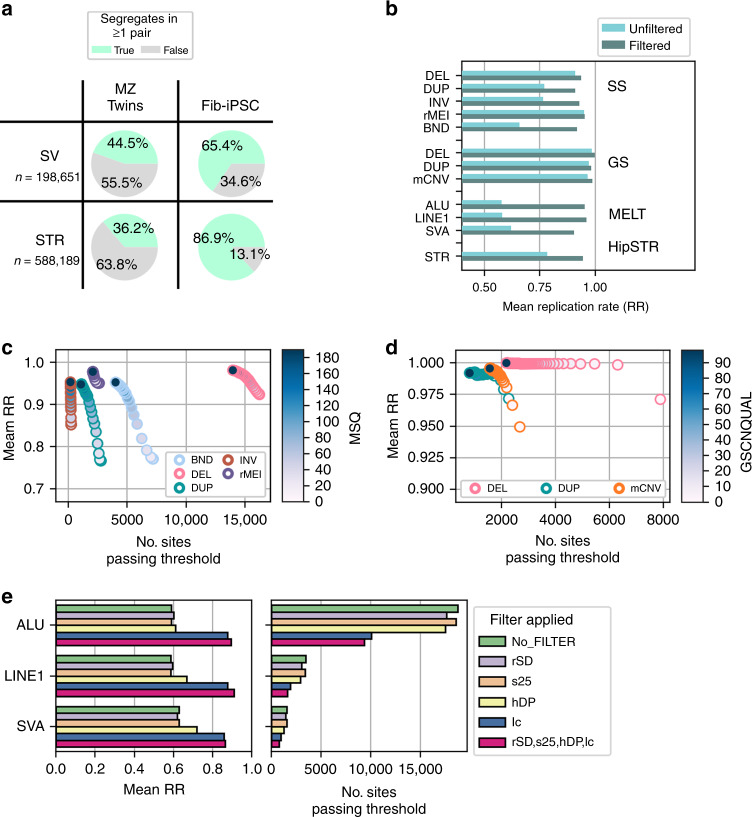
Replication rate is associated with reported quality metrics. **a** Proportion of SVs and STRs that were non-reference (green) in at least one of the iPSCORE MZ twin pairs or HipSci fibroblast–iPSC pairs prior to filtering. **b** Replication rate of variants before and after filtering and deduplicating within caller; Genome STRiP and SpeedSeq abbreviated in figure as GS and SS respectively. **c** Replication rate in MZ twins versus the number of total SpeedSeq (LUMPY) sites remaining that pass criteria when filtering variants to different thresholds for MSQ score (indicated by color). **d** Replication rate versus the number of total Genome STRiP sites remaining that pass criteria when filtering variants to different thresholds for GSCNQUAL score (indicated by color). **e** Replication rate in MZ twins for MELT sites that pass criteria when filtering variants under suggested hard site filters (left). Pink represents the result of filtering using all four exclusion criteria (rSD, s25, hDP, lc; see Methods). The number of total sites remaining that passed criteria is shown at right.

Because there is considerable diversity in subtypes of SVs and disparities between detection algorithms, measuring SV quality is challenging. Here we used 177 genetic replicates (25 MZ twin pairs and 152 matched fibroblast and iPSC pairs) to measure reproducibility of SV calls for each variant class and SV calling approach under a range of quality metric filter thresholds. Because of complications in variant calling on sex chromosomes due to dosage differences in males and females, we analyzed reproducibility among 198,651 autosomal SVs. Notably, we were able to assess the reproducibility of most variants in the SV call set since 44% of autosomal SVs (88,496) segregated in at least one monozygotic twin pair, 65.4% (129,937) segregated in at least one fibroblast–iPSC pair (Fig. 2a), and 71.8% (142,678) segregated in any of the 177 genetic replicates. For each variant that segregated in at least one genetic replicate pair, we assessed reproducibility by calculating how often a non-reference genotype in one replicate pair sample was called concordantly in the other replicate sample, which we define as replication rate (RR, Methods). Replication rates were calculated for each SV separately among MZ twin pairs and fibroblast–iPSC pairs. The 25 MZ twin pairs were used to select filters because they have matched cell types and fewer somatic differences^39^ while the 152 matched fibroblasts–iPSC pairs were used to confirm the performance of these thresholds in the HipSci collection. While filtering is expected to result in improved reproducibility, using these genetically matched sample pairs we were able to explicitly quantify differences in reproducibility between different variant classes and methods.

Prior to filtering on quality metrics (Supplementary Table 1), we observed that within the 25 MZ twin pairs CNVs (deletions, duplications and mCNVs) detected with Genome STRiP showed high reproducibility (RR > 0.96) as did the SpeedSeq deletions (RR > 0.9) and rMEIs (RR > 0.95), whereas SpeedSeq duplications and inversions (both RR < 0.77), BND (RR = 0.65), and MELT MEIs (RR = 0.59) had lower reproducibility (Fig. 2b). We found that for all variant callers, increasingly strict quality metric filters yielded variant sets with higher average RRs, supporting the premise that reproducibility is a predictor of variant quality (Fig. 2c–e). For example, we found strong relationships between Median Sample Quality (MSQ) score from SpeedSeq, the GSCNQUAL score from Genome STRiP, and qualitative filters from MELT and the average RR of filtered variants (Fig. 2c–e). Notably, filtering MELT variants called in low-complexity regions (lc tag in FILTER) improved reproducibility from 59% to 87.5% in MZ twins and applying all four MELT filters improved RR to ~95% (Fig. 2e, Methods). Using RR we selected strict quality metric thresholds for each caller and variant class to achieve high specificity without removing a significant number of variants. We observed that within each algorithm, different variant classes required different levels of filtering stringency to attain the same reproducibility (Fig. 2c, d). For instance, insertions and duplications were less reliably genotyped than deletions regardless of detection method^19,21,32^, and SpeedSeq duplications required an MSQ score of 100 to attain >0.9 RR while deletions had an RR of 0.92 with no MSQ filtering (Fig. 2c) in MZ twin pairs.

After filtering, we obtained 50,980 autosomal variants (20.2% of initial call set) with generally high RR (>0.9) for all callers, although variants called by SpeedSeq and MELT tended to have lower RR than those called by Genome STRiP (Fig. 2b), suggesting that variants called using read-pair signal are less reproducibly genotyped between genetic replicates than than those called by read-depth signals. We tested for batch effects by comparing allele frequencies between iPSCORE and HipSci samples and found that they largely agreed across algorithms (Supplementary Figs. 2, 3 and 6). We compared the CNV genotypes to those called from SNP arrays for 216 iPSCORE samples and found that the false discovery rate (FDR) for CNVs ranged from 3% to 7.8% depending on the SV type and algorithm, consistent with previous reports^18,19^. We also found that biallelic SVs generally obeyed Hardy Weinberg across algorithms after filtering (Supplementary Figs. 2, 3 and 6). Together, these results suggest that our stringent filtering approach can be used to obtain comparable, high confidence variants across SV classes and algorithms.

### Creating a high confidence, non-redundant SV call set

**Fig. 3:**
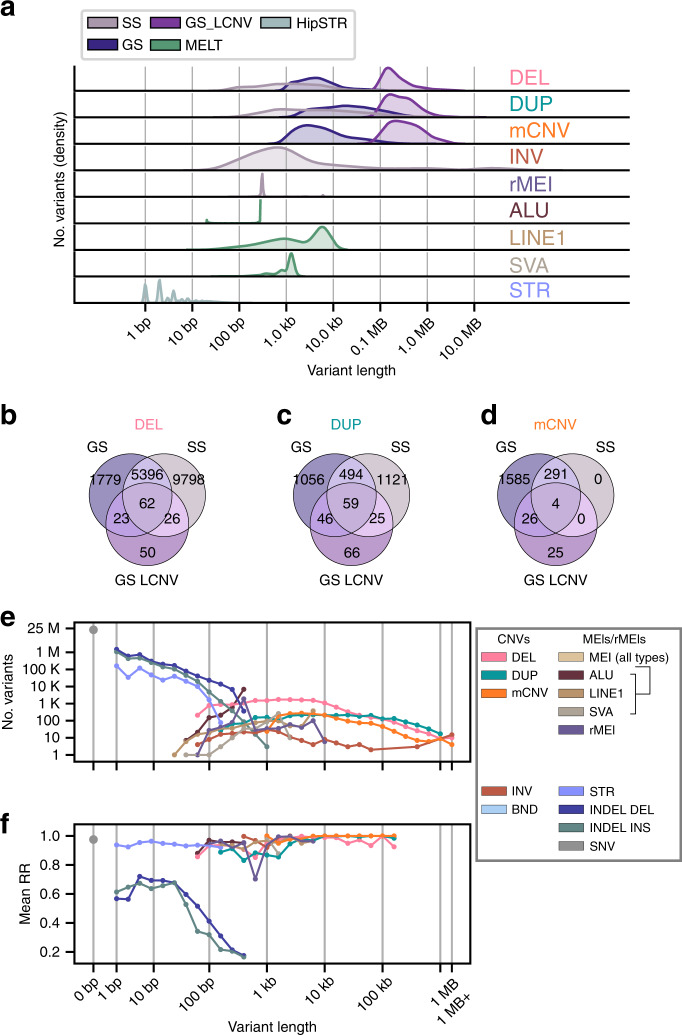
Variant length distributions and variant caller comparison. **a** Density plot showing the size spectrum of each variant caller before identifying multi-caller clusters. **b**–**d** Number of overlapping variants after identifying multi-caller clusters for deletions (**b**), duplications (**c**), and mCNVs (**d**). **e** Number of variants in the non-redundant call set separated by variant class and grouped in log linear bins by variant length. Points are drawn at the upper limit of each bin (e.g. a bin from 50 to 100 bp is drawn at 100 bp). For STRs length represents the maximum number of bases different from the reference at each site (largest insertion or deletion observed). **f** The average replication rate of variants segregating in the 25 monozygotic twin pairs is represented for each length bin that contains at least 10 variants. GATK SNVs and indels previously discovered in iPSCORE samples^40^ were used for **e** and **f**.

SV calling algorithms overlap in the types and sizes of variants they identify (Fig. 3a) which can lead to the same genetic variant being called with slightly different breakpoints by different algorithms in the same subject or by the same algorithm in different subjects. To obtain a non-redundant map of structural variation, we devised a graph-based approach to consolidate overlapping sites that are redundant with each other (Supplementary Figs. 7 and 8, Methods). We first clustered overlapping variants that were detected using the same algorithm and showed high genotype correlation and designated each cluster as a single distinct SV with a breakpoint defined by the highest quality variant (Fig. 1a). We next stitched together neighboring variants from Genome STRiP whose genotypes were correlated because they likely represent a single variant that Genome STRiP called as multiple adjacent variants^19^. Finally we clustered overlapping variants identified by different algorithms with high genotype correlation and designated each multi-caller cluster as a single distinct SV (Fig. 3b–d, Methods). We inspected variants identified by multiple algorithms and found that overlap between Genome STRiP and SpeedSeq was highest among deletions (55%), while duplications and mCNVs were only co-discovered 17% and 15% of the time, respectively, reflecting both the different size spectrums captured by the two methods (SpeedSeq captures smaller variants) and that evidence types (read-pair/read depth) do not always co-occur. SVs identified by more than one algorithm (i.e. with support from both read-pair and read-depth signals) had higher RRs than SVs detected with a single algorithm (Supplementary Fig. 9), supporting the premise that the highest quality sites also tend to be the most reproducible. Overall, we collapsed 50,980 variants to 37,296 non-redundant SVs which were used for downstream analyses (Table 1, Supplementary Data 3, Supplementary Figs. 10 and 11). We examined the numbers and proportions of non-reference calls for each of the 719 i2QTL samples (from 477 individuals) across variant calling algorithms and variant classes (Supplementary Figs. 2, 3, 5 and 6). We observed high consistency in the number of variants per sample except for individuals with African ancestry who had more SVs per sample, consistent with other variant types^18,48^. Taken together, these results show that the set of i2QTL SVs consists of high confidence variants and demonstrates the utility of using genetic replicate samples for SV filtering and processing.

**Table 1:** Summary of i2QTL variants called from samples in the HipSci and iPSCORE collections.

Variant class	No. of variants	No. of common variants
SNV	41,826,418	7,013,178
INDEL	7,040,457	1,862,365
Deletion (DEL)	16,238	3,490
Duplication (DUP)	2693	416
Multiallelic CNV (mCNV)	1703	949
Other SV (BND)	4612	1,377
Inversion (INV)	210	92
Reference mobile element insertions (rMEI)	2343	1689
ALU	7880	2385
LINE1	1175	262
SVA	442	115
Short tandem repeats (STR)	588,189	381,053
Total SV	37,296	10,775
Total SV/STR	625,485	391,828
Total	49,492,360	9,267,371

Common variants are defined as those with ≥5% non-mode allele frequency (NMAF) for SVs and STRs and ≥5% MAF for SNVs and indels.

### Variant length, allele frequency, and reproducibility

Since SVs can vary widely in size and we are using short read data to call SVs, we assessed whether RR was related to SV length. While we could detect many more short SVs (<1 kb) than long SVs, we observed that long SVs had higher RR (Fig. 3e, f). Generally, SVs greater than 1 kb were highly reproducible (>95% RR) while shorter duplications and insertions tended to have the lowest RR, reflecting the relative lack of consistency in genotyping small read-pair-based SVs. This dependence on length was observed across variant calling approaches and independent of allele frequency (Supplementary Fig. 12a, c). We also found that rare variants were slightly less reproducible than common variants across SV classes (Supplementary Fig. 12b, d). These results highlight that it remains challenging to identify SVs in intermediate size ranges (~200 bp to 1 kb) using short read sequencing, because the interval is: (1) too small to distinguish from noise in read-depth signal; (2) within the bounds of variability in insert size, making discordant read-signal undetectable; and (3) too long to be directly sequenced with a single read. While challenges in the discovery of SVs in the ~200 bp to 1 kb range still exist, the i2QTL call set consists of high-quality SVs across a wide size range of SVs (~50 bp to >1 Mb).

### Comparison between SVs in i2QTL and other SV resources

**Fig. 4:**
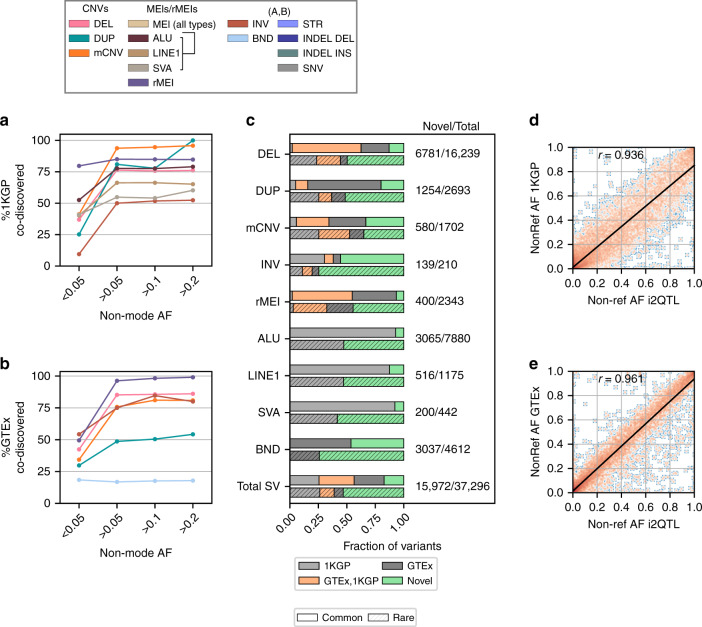
Comparison to other SV calling studies. **a**, **b** The fraction of variants from either **a** 1KGP (European population) or **b** GTEx that were also captured in our study in different non-mode allele frequency (NMAF) bins. **c** Fraction of i2QTL SVs that were co-discovered in 1KGP, GTEx, both 1KGP and GTEx, or were unique to i2QTL (novel), divided by whether variants were common (>0.05 NMAF) or rare (<0.05 NMAF) in unrelated i2QTL samples indicated by absence or presence of hatching respectively. **d**, **e** Non-reference allele frequency of variants co-discovered in i2QTL and **d** 1KGP (Europeans) or **e** GTEx in their respective discovery samples. Here, the non-reference allele frequency among unrelated i2QTL donors is used, and the density is plotted with orange indicating more observations, and blue fewer.

We next investigated what proportion of the SVs in the i2QTL call set are novel compared to previous SV call sets by comparing the 37,296 non-redundant i2QTL SVs with the 1KGP^18^ and GTEx^19^ SV call sets. GTEx used 148 deeply sequenced genomes and the 1KGP project used 2504 shallowly sequenced genomes (7.4×) to call the same SV classes present in i2QTL (excluding BND in 1KGP and non-reference MEIs in GTEx) and are therefore strong benchmark datasets. The i2QTL SV call set captured the vast majority of common deletions, duplications, mCNVs, inversions, rMEIs, and MEIs present with non-mode allele frequency (NMAF) greater than 0.05 in either study, including 77% of variants present in 1KGP Europeans and 79% of variants present in GTEx (Fig. 4a, b). Out of all SV classes, we captured the smallest proportion of common GTEx duplications (49%) and BND (17%) likely due to differences in filtering stringency, WGS data quality, and breakpoint merging approaches. In total, 83% of common i2QTL SVs (NMAF > 0.05) were co-discovered by one or both of these studies (Fig. 4c). Common deletions had the highest co-discovery rate (87%) while mCNVs had the lowest (~66%), consistent with the idea that mCNV discovery benefits from high read-depth and large numbers of samples^29^. Rare variants (NMAF < 0.05) were more likely to be unique to either set, with ~40% of sites from GTEx and 1KGP represented in the i2QTL call set (Fig. 4c). In total, 43% of i2QTL SVs were not found in either GTEx or 1KGP. These novel variants were predominantly rare, tended to have shorter lengths, and, excluding those identified by Genome STRiP, had on average 12% lower RRs than co-discovered variants (Fig. 4c, Supplementary Figs. 13–15). This is expected given that small SVs are the most difficult to genotype and rare variants are more likely to be false positives or negatives (Supplementary Fig. 12). To assess how similar genotyping sensitivity was between studies and confirm that overlapping sites were likely to have the same breakpoint, we compared the non-reference allele frequencies of sites that we classified as co-discovered. We found that overall the non-reference allele frequencies of i2QTL variants were highly correlated (*r* > 0.9) with their matched GTEx and 1KGP variants (Fig. 4d, e). This was true across variant classes in both studies, with the exception of duplications in 1KGP, which were less correlated (*r* = 0.75), likely as a result of limited genotyping sensitivity in 1KGP due to the use of low coverage WGS data (Supplementary Figs. 16 and 17). Overall the i2QTL call set contains a significant number of novel, high-quality variants at lower allele frequencies missing from 1KGP and GTEx.

We also compared i2QTL variants to the recently released SV call set generated from high-coverage short read WGS data from 14,891 individuals from the gnomAD consortium^49^ (Supplementary Fig. 18). We found that the i2QTL SV call set captured a large fraction of common (>0.05 minor allele frequency (MAF)) deletions, insertions, and mCNVs found in European individuals from gnomAD SV (46%, 32%, and 34% respectively) while biallelic duplications were the least represented (15%). Poor overlap with biallelic duplications was found to be driven largely by gnomAD containing small duplications not present in i2QTL (Supplementary Fig. 18c, d), and was likely due to differences in merging and filtering strategies between the two studies. Taken with the comparisons to GTEx, and 1KGP, these results suggest that despite a relatively small sample size, the i2QTL call set contains a large fraction of SVs in Europeans that are discoverable with short read sequencing.

In addition to short read WGS variant call sets, there have been recent efforts from consortia such as Genome in a Bottle (GIAB) to create benchmark samples for SV calling that make use of long read sequencing technology^50,51^. To investigate how well variants captured by longer reads are represented in the i2QTL SV dataset, we repeated variant calling on a set of 75 iPSCORE samples, and included GIAB/1KGP sample NA12878 downsampled to 48× coverage to match the median coverage of iPSCORE samples. This sample has the advantage of being included in the 1KGP SV call set as well as having SVs obtained from PacBio sequencing. Comparing SVs discovered with our methods for NA12878 to those from the short read SV call set from 1KGP for this sample, we co-identify a large majority of variants across all classes (total: 2637/3253, 81.1%, DEL 78.7%, DUP 75%, mCNV 62%, MEI 76%, rMEI 98.4%) (Supplementary Fig. 19). Comparing to the PacBio SVs called for this sample, we find 2474/4495 (55%) deletions, 8/243 (2.8%) duplications, and 671/5815 (11.5%) insertions (Supplementary Fig. 20a, b). Variants that were unique to PacBio sequencing tended to be small (50 bp–1 kb) while variants unique to i2QTL tended to be longer (1–10 kb) (Supplementary Fig. 20c, d). These results, along with the poor reproducibility of variants in the 50 bp–1 kb size range (Fig. 3e), suggest that small SVs remain difficult to discover with short reads alone.

We next sought to determine whether some of the small duplications discovered in gnomAD but not i2QTL might be identified in i2QTL by using additional variant calling methods. To do so, we ran wham^25^ and manta^52^ on NA12878 and the 75 iPSCORE samples described above. We collapsed the overlapping variants from wham and manta (Supplementary Figs. 21 and S22, Methods) resulting in 16,681 non-redundant SVs (7656 deletions, 1986 biallelic duplications, 1798 rMEI, 760 mCNV, 4139 MEI, and 342 inversions). Of these 16,681 SVs, 2930 (17.56%) were attributable exclusively to wham or manta (or were discovered by both callers) (Supplementary Fig. 22). We intersected this combined variant call set for the 75 iPSCORE samples plus NA12878 with gnomAD SVs to determine the proportion of variants co-discovered considering the additional variant callers. Interestingly, while variants unique to SpeedSeq and Genome STRiP were largely co-discovered by gnomAD (58.5% and 46.8%, respectively), variants unique to manta and wham were rarely co-discovered by gnomAD (10.2% and 9.45%, respectively), suggesting that these approaches may have a higher FDR. Furthermore, the gnomAD variants that wham and manta uniquely identified in the 75 iPSCORE samples were overwhelmingly deletions (429/583 SVs, 83.6%) rather than the small duplications that are missing from i2QTL relative to gnomAD. These results indicate that the i2QTL SV call set contains a large fraction of common SVs in Europeans discoverable using short read sequencing data as well as new, rare SVs, making it a valuable resource for examining functional differences between the SV classes^53^.

### STR genotyping

We genotyped STR variants at over 1.6 million reference sites using HipSTR^38^, which employs a hidden Markov model to realign reads around each STR locus (Fig. 1a). HipSTR models PCR stutter artifacts to genotype STRs and because of such artifacts, greater genotyping sensitivity and accuracy of predicted de novo STR alleles can be achieved with PCR-free WGS data. In light of this, HipSci samples, which were generated with a PCR-free library preparation, were genotyped separately and then these alleles were used as a reference to genotype iPSCORE samples, which were prepared using a PCR-based library prep, and the results for both sample sets were combined into one call set with consistent alleles. To retain only high-quality STR calls, we applied the genotype specific filters suggested by HipSTR^38^ and required all sites to have an 80% call rate in iPSCORE or HipSci samples. This resulted in 588,189 autosomal variants with high reproducibility across the range of gentotyped expansion/deletion sizes (1–150 bp) (overall 94.5%, >90% in all size bins); these variants were substantially more reproducible than indels in this same size range called by GATK in the i2QTL call set, which overall showed low RRs (62%) (Fig. 3e, f, Supplementary Figs. 23 and 24). In total, 231,317 of the 588,189 STRs (39.3%) had four or more observed length alleles and could be classified as multiallelic. Because HipSTR STRs and GATK indels overlap in size and location, it is likely that some variants are present in both datasets. To compare the genotyping quality of these possibly redundant variants, we intersected GATK indels with 1.6 million HipSTR STR reference loci (Supplementary Fig. 12e). Interestingly, we found that indels (2–100 bp) called by GATK that overlapped an STR locus that was genotyped non-reference in at least one sample by HipSTR had higher RR (77.3%) than those that overlapped STR loci not genotyped as polymorphic by HipSTR (56%), or those that did not overlap an STR region (64.7%). These findings suggest that it is useful to filter large GATK indels (>30 bp) because they have low RR (42%), and that STR genotypes are more reproducible than GATK indels.

### Linkage disequilibrium tagging for SVs and STRs

**Fig. 5:**
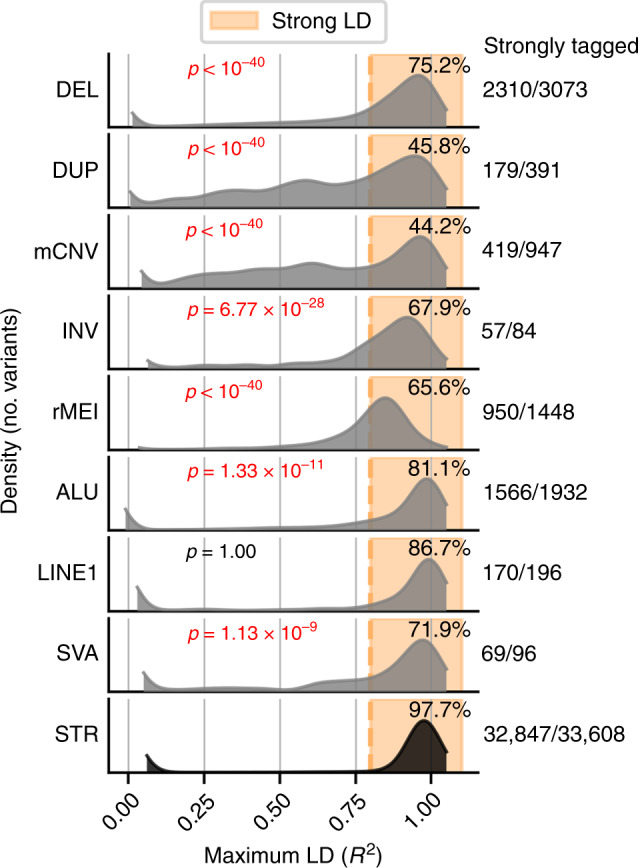
Linkage disequilibrium tagging of structural variants and short tandem repeats. Distribution of maximum linkage disequilibrium (*R*^2^) in i2QTL Europeans between common SVs and STRs (non-mode allele frequency > 0.05) and SNVs or indels within 50 kb, considering only SVs/STRs that are within 1 MB of an expressed gene in iPSCs.

Given the large amount of GWAS and QTL studies performed using genotyping arrays, we next asked to what extent different classes of SVs and STRs are tagged by SNPs and indels. For each of the 42,921 common (NMAF > 0.05) SVs and STRs that were within 1 MB of an expressed gene, we calculated the maximum linkage disequilibrium (LD) in i2QTL Europeans with SNPs/indels within 50 kb (Methods)^53^. We found that 97.7% of STRs are tagged by an SNP or indel with *R*^2^ > 0.8 while SVs classes ranged from 44.2% to 86.7% of variants tagged with *R*^2^ > 0.8 (Fig. 5). Duplications and mCNVs were the most poorly tagged classes likely because they are often located near segmental duplications where SNPs and indels are poorly genotyped^18,29,33^. These results indicate that most common STRs and some classes of SVs are assayed well by proxy using SNP and indel genotypes, but to increase the coverage of SVs, particularly mCNVs and duplications, studies need to include the genotyping of these variant classes in their samples.

## Discussion

In this study, we discovered and genotyped SVs and STRs in 719 high-coverage WGS samples from 477 unique donors. We detected a wide spectrum of variants across different sizes as most STRs are in the 10 bp to 1 kb range whereas SVs may span more than 100 kb. We leveraged genetic replicates, such as twin pairs and fibroblast–iPSC matched samples, to test variant calling accuracy and determine filtering approaches to retain only high-quality SVs and STRs. Our filtered call set has very high RR, indicating high genotype quality for detected SVs and STRs. The call set captures most of the common variants identified in 1KGP^18^ and GTEx SV variant calling efforts and also contributes novel short (~100–1000 bp) and rare (NMAF < 5%) variants. The high confidence, non-redundant i2QTL SV set described here will serve as a useful reference for other studies and is particularly valuable for genetic association analyses that aim to identify SVs that influence disease risk or quantitative molecular traits like gene expression.

We used five algorithms designed for calling variants across many samples to detect different classes of SVs and STRs and compared the RR in genetic replicates (MZ twin pairs and fibroblast–iPSC pairs) to identify factors that impact RR. We found that we needed to call variants separately in the iPSCORE and HipSci WGS collections and implement specific filtering strategies to account for dataset-specific features such as library preparation techniques to achieve high RR. Given the variability in library preparation methods, future improvements to SV calling algorithms may explicitly adjust for specific library features such as PCR-free sequencing. We also observed differences in RR between different classes and sizes of SVs and different algorithms. We found that SVs in the 100–1000 bp range remain harder to identify and genotype likely due to the limitation of using short reads. We also observed that accuracy was highest for large (>10 kb) duplications, deletions, and mCNVs suggesting that FDR estimates from orthogonal datasets such as arrays may overestimate accuracy for SV call sets since they generally assess the largest and easiest-to-genotype variants. Future studies that combine deep short read WGS with long read sequencing data may be able to improve the detection and genotyping of SVs in the 100–1000 bp range by directly sequencing them or assembling the short and long reads.

We used genetic replicates to identify algorithm- and SV-specific thresholds and applied these thresholds to filter the initial set of SV calls and create a high confidence catalog of SVs and STRs that complements previous SVs identified using low depth WGS or fewer samples^18,19^. We also developed approaches for collapsing redundant SVs and harmonizing SVs called by different algorithms across hundreds of samples. Comparing our SV catalog to previous sets of SVs from the 1KGP and GTEx projects shows that the i2QTL SV call set captures most common (NMAF > 0.05%) SVs in Europeans. However, consistent with other types of genetic variants, we found that African ancestry samples had more SVs than Europeans. Future sequencing studies are needed to fully catalog SVs in other ancestries and identify rare, population-specific SVs. Such multi-ancestry SV catalogs will be indispensable for population sequencing studies such as All of Us^54^ that aim to integrate genetic and health data for patients from diverse and admixed ancestries.

The filtering scheme and catalog of SVs and STRs presented here can be used in future genetic association and sequencing studies that aim to study the impact of SVs/STRs. One method for utilizing this catalog for calling SVs and STRs is to impute variants via tagging SNPs and indels; a benefit of this approach is that imputation is possible using both array- and sequenced-based genotyping. A second option when sequencing data is available is to skip the de novo SV and STR discovery step and instead genotype the reproducible variants reported here. This will restrict genotyping to high-quality sites and may lessen the burden of filtering variant calls. A third option is to perform de novo discovery, genotyping, processing, and filtering using the approaches and thresholds that we have identified. While it may be possible that some filtering thresholds need to be adjusted for specific studies, the thresholds provided here likely provide a good starting point for genotyping and filtering de novo discovered SVs and STRs in other datasets.

Overall, this study provides a roadmap for discovering and genotyping SVs from WGS data and establishes a high-quality catalog of SVs and STRs that can be used in future genotyping efforts. A companion paper^53^ examines how the i2QTL SVs and STRs characterized here influence gene expression and contribute to disease risk. These studies demonstrate that SVs and STRs can be reliably identified and genotyped for hundreds of samples and used to study the impact of this class of genetic variation on human health.

## Methods

### Subject enrollment

In total, 273 subjects were recruited as part of the iPSCORE study, of which 215 subjects have been included in previous studies^39–41^. Data for additional 204 subjects were obtained from the HipSci Collection^42,43^. We have complied with all relevant ethical regulations for work with human participants and obtained informed consent. The iPSCORE collection was approved by the Institutional Review Board of the University of California at San Diego (Project #110776ZF). Each of the subjects provided consent, filled out a questionnaire, had blood drawn, and had a 1 mm skin biopsy taken from which fibroblasts were obtained. Five individuals provided consent only for cardiovascular studies; therefore, they were removed from downstream analyses. Family relatedness, sex, age, and ethnicity were recorded in the questionnaire. Detailed pedigree information for iPSCORE available in Panopoulos et al.^39–41^ (dbGAP: phs001035). In total, we utilized a total of 477 HipSci and iPSCORE subjects, 276 were females and 201 were males, and collectively subjects ranged in age from 5 and 89 years of age (Supplementary Fig. 1a). Notably, iPSCORE individuals were included in 56 families composed of two or more subjects (range: 2–14 subjects) and 86 single individuals (Supplementary Fig. 1b, Supplementary Data 1). Overall, 167 iPSCORE individuals were unrelated. All iPSCORE individuals were grouped into one of five superpopulations (European, African, Admixed American, East Asian, and South Asian) on the basis of genotype data^39–41^ and HipSci samples were similarly categorized^42^ (Supplementary Fig. 1c). For HipSci, some subjects had multiple iPSC clones with WGS. For these subjects, we chose the pair of fibroblast and iPSC WGS samples that had the highest reproducibility for Genome STRiP calls (see Genome STRiP CNVDiscovery RR analysis).

### WGS data processing

*IPSCORE:* WGS sequencing for iPSCORE individuals is available on dbGaP (dbGAP: phs001035)^40^. DNA isolated from either blood (254 samples) or fibroblasts (19 samples) (Supplementary Data 2, Fig. 1a) was PCR-amplified and sequenced on Illumina HiSeqX (150 base paired end). We obtained an average of 180.9 billion total raw bases per sample (range 117.81–523.49 billion bases). The quality of raw fastq files was assessed using FASTQC^55^. Reads were then aligned to the human b37 genome assembly with decoy sequences included and a Sendai virus contig with the BWA-mem algorithm under default parameters^56^.

*HipSci:* We downloaded cram files associated with 446 genomes (mean depth 36.3×) generated with a PCR-free protocol from 204 healthy donors (ENA Study Accession: ERA828)^42^. Genomes were aligned to hs37d5 genome, a reference identical to the one used for iPSCORE alignments with the exception of the inclusion of a Sendai virus contig. Cram files were converted to the bam file format and merged when necessary using samtools^57^.

Bam files from both iPSCORE and HipSci were sorted with sambamba^58^ and duplicates were marked with biobambam2.

### Variant callers and types of genetic variants detected

We used five variants callers to identify SVs and STRs. We used the SpeedSeq (SS) SV pipeline^46^ that combines LUMPY^24^ read-pair evidence with read-depth support from CNVnator^26^. We also used the Genome STRiP CNVDiscovery pipeline (GS) and Genome STRiP LCNVDiscovery pipeline (GS LCNV)^29^ that detect SVs based on read-depth evidence. We used MELT^47^ for mobile element insertion discovery and HipSTR^38^ to identify and genotype STRs. LUMPY, GS, and GS LCNV each identified biallelic deletions (DEL), biallelic duplications (DUP), and mCNVs. mCNVs are defined as variants that have at least three predicted alleles. LUMPY identified inversions (INV) and generic BND that can include deletions and duplications that lack read-depth evidence, balanced rearrangements (INVs), MEIs, or other uncategorized breakpoints. As part of the SpeedSeq pipeline we also identified reference mobile elements (rMEIs). For non-reference MEIs we used MELT to identify Alu element insertions (ALU), LINE1 element insertions (LINE1), and SINE-R/VNTR/Alu element insertions (SVA). HipSTR identifies STRs where at least one individual differed in STR length compared to the reference. We considered CNVs to include deletions, duplications, and mCNVs. We considered MEI to encompass non-reference MEI ascertained by MELT, including ALU, LINE1, and SVA elements.

### Replication rate and filtering strategy for SVs and STRs

To minimize the number of poorly genotyped SVs and maximize quality across multiple variant calling approaches, we used the RR metric, calculated as the proportion of non-reference genotypes that were also called non-reference in a paired genetic replicate, as a measure of the reproducibility (and quality) of a variant. The rationale behind this approach is that variants that have high genotyping accuracy should be genotyped consistently in different samples with the same genome and that variants with low genotyping accuracy will differ between samples with the same genome. Under this logic, variants should be consistently genotyped in samples with the same genomes (e.g. technical duplicates, monozygotic twins) and discrepancies would result from false-negative or false-positive genotypes.

To determine RR for all variant classes, we used genetic duplicate samples in the form of monozygotic twin pairs (*n* = 25) and fibroblast–iPSC pairs (*n* = 152). We used RR to assess the reproducibility of variants under different filtering conditions; the filters were specific to the unique quality metrics measured by each calling algorithm. Using the relationships between filters and RR that we identified, we selected filtering criteria for each variant class in each caller to maximize the quality (specificity) and the number of variants (sensitivity) called. Because there may be a greater number of somatic variations between fibroblasts and iPSC clones^39^ due to reprogramming, RRs in monozygotic twins were used to select thresholds, and iPSC–fibroblast pairs were used for additional confirmation. For this analysis, one member of each pair of genetic duplicates was chosen arbitrarily as the comparison sample, and the concordance of non-reference sites in this sample was assessed with respect to the other sample. The replication rate was calculated on all autosomal SVs on a site-by-site basis as the number of pairs with matching non-reference genotypes divided by the total number of pairs with at least one non-reference genotype. Average RRs reported for particular SV classes were calculated as the average RR over all SVs in that class.

### Batch effects and Hardy–Weinberg equilibrium filtering

The i2QTL Consortium includes WGS data from iPSCORE and HipSci^41,42^, which are different in aspects which may affect variant calling: (1) mean coverage is higher for iPSCORE (50.4×, compared with 36.6×); (2) while most iPSCORE donors had WGS from blood and only 14 from skin fibroblasts, all HipSci donors had WGS from skin fibroblasts; and (3) HipSci samples were sequenced using a PCR-free protocol (Supplementary Fig. 1, Fig. 1, Supplementary Data 2). To limit the batch effects associated with these differences, in cases where a variant caller used information from the entire set of samples to build a global model (Genome STRiP^29^ and HipSTR^38^), we genotyped or performed discovery separately in iPSCORE samples and HipSci samples, which were additionally divided into two groups for fibroblast and iPSC samples.

We compared allele distributions for autosomal variants ascertained for unrelated members of each collection (167 unrelated iPSCORE samples and 204 HipSci samples) after variant calling and filtering to ensure that differences between WGS from each collection did not create widespread systematic artifacts in variant calling. Allele distributions were compared between the studies using a chi-squared test with a Bonferroni correction. For instance, for an insertion, the number of samples with zero, one, or two copies of the insertion in iPSCORE were compared to the number of samples with zero, one, or two copies of the insertion in HipSci using the chi-squared test. Variants with Bonferroni-corrected *p* < 0.05 were tagged in the VCF file. For this analysis, missing genotypes were also included as a unique allele when present.

We calculated Hardy–Weinberg equilibrium to identify variants that could be affected by batch effects in variant calling or that were poor quality. We used all unrelated blood/fibroblast samples and considered autosomal biallelic duplications and deletions from Genome STRiP^29^ as well as all variant classes ascertained by SpeedSeq^46^ and MELT^47^. We tested HWE using a chi-squared test to compare the counts of the observed genotypes to those expected given HWE. SVs with Bonferroni-corrected HWE *p* < 0.05 were flagged as potentially not obeying HWE.

### Filtering based on number of calls ascertained per sample

Consistency in the number of non-reference calls per sample is associated with variant calls from high-quality WGS sequencing data, samples of similar ancestry, and algorithm performance. We counted the number of calls per sample for all algorithms to assess whether there were differences in the number of SVs identified in samples from each study, ancestry, or cell type from which the WGS was derived.

### SpeedSeq variant calling

We used the split and discordant read-pair-based structural variant caller LUMPY (v0.2.13)^24^ under its implementation in SpeedSeq (v0.1.2)^46^ to call duplications, deletions, inversions, and other novel adjacencies referred to as BND. We ran LUMPY on each of 719 samples (478 from the HipSci collection and 478 from the iPSCORE collection) using the speedseq sv command with the -P option to retain probability curves in the output VCFs, -d to CNVnator (v0.3.3)^26^ to calculate absolute copy number information on each sample, and -x to exclude a published list of genomic regions (ceph18.b37.lumpy.exclude.2014-01-15.bed) known to be potentially misassembled regions^24,59^. Calls from individual samples were then genotyped using SVTyper (v0.1.4), before being combined into a single VCF file. Individual VCF files were sorted, and merged using svtools (v0.3.2) with the sort and merge command (slop 20 bp) to remove overlapping breakpoints, resulting in a single VCF file with the most probable sites. Each sample was then genotyped at these merged sites using SVTyper and annotated with an absolute copy number using the svtools copynumber command. Variants were merged into a single VCF file, pruned, and reclassified under suggested parameters^19^. Individual VCFs were merged using svtools vcfpaste and further processed to remove additional identical variants using svtools prune. This set of breakpoints was then reclassified by using svtools classify to identify high confidence CNVs by regressing the estimated copy number and allele balance information (non-reference/reference reads at an SV site) as well as to identify MEIs in the reference genome (rMEI, which appear as deletions in our call set).

### SpeedSeq variant processing

Because metrics such as RR may select variants that are reproducible artifacts, to remove as many known low-quality sites as possible, we first applied suggested filtering guidelines^19^ as follows: (1) deletions that were less than 418 bp were required to have split read support; (2) all non-BND variants were required to be at least 50 bp in length; (3) BND calls required 25% support from either split or paired-end reads; and (4) QUAL > 100 inversions were required to have at least 10% of evidence from split or paired-end reads. Finally, to ensure a baseline level of genotyping consistency at each site, variants were filtered if they had a missing rate of >10%.

### SpeedSeq variant redundancy collapsing

After running the Speedseq/SVtools pipelines and filtering variants as described above, the variant call set still contained overlapping variants suspected to be identical. To produce a single set of non-overlapping unique calls, we performed additional pruning steps. To identify and prune putatively identical calls that remained in our call set we implemented a graph-based approach: (1) we constructed a graph where SVs with reciprocal overlap of at least 50% are nodes connected by an edge; (2) we created a correlation matrix for each set of connected components using the allele balance (non-reference/reference reads at an SV site) at each site across individuals; (3) we refined the graph, retaining only the edges between SVs with *r* > 0.25 at a given site, which are likely to represent a single breakpoint; (4) we iterated through connected components, and chose variants with the highest MSQ score, pruning other variants in the subgraph; and (5) in cases where one call was fully contained within another call and there was a correlation of at least 0.5 in allele balance between them, indicating that both calls were genotyped as non-reference in the same individual(s), only the site with the highest MSQ score was retained.

### SpeedSeq RR analysis and filter selection

During SpeedSeq quality analysis we investigated supporting reads (SU) and MSQ as possible filtering criteria and found that MSQ was strongly associated with RR in iPSCORE twins and HipSci fibroblast–iPSC pairs (Supplementary Fig. 2a, b) while the number of supporting reads was not. For variant filtering, we determined variant class-specific MSQ thresholds, with the goal of ensuring at least 90% RR across all variant classes and retaining the maximal number of variants. Classes of variation that were highly reproducible before quality score filtering (>90% RR) were filtered at a 20 MSQ score^19^. With this approach, we performed additional filtering as follows: (1) deletions and rMEIs must have MSQ > 20; (2) duplications, inversions, and BND calls must have MSQ > 100, 90, and 90, respectively. Deletions and rMEIs were genotyped most reproducibly, prior to filtering, while duplications were less reliably genotyped, reflecting the sensitivity of split read versus discordant read signal. After filtering, RR was on average 97% in twin pairs and slightly worse (92%) in fibroblasts–iPSC pairs (Supplementary Fig. 2c).

### SpeedSeq batch effect and Hardy–Weinberg analysis

We tested variants on autosomes that passed the filters described above and 196 variants with missing rate >10% but that otherwise passed filters for differences in allele distribution or deviations from HWE as described above (see Batch effects and Hardy–Weinberg equilibrium filtering). We found that only 544 of 25,537 sites tested had different allele distributions (2.1%, Supplementary Fig. 2d). We also observed that 1256 variants (4.9%) deviated from HWE, suggesting that batch effects do not affect SpeedSeq variant calls. We also observed that allele frequencies were highly correlated between variants detected in iPSCORE and HipSci.

### SpeedSeq calls per sample

After variant calling, we found that the number of SVs identified was consistent across samples, regardless of the study or cell type (Supplementary Fig. 2e, f). In agreement with previous SV discovery studies, we observed on average 10.2% more SpeedSeq variants per sample for those of African ancestry (4260/sample)^18,19^ as compared to samples that were not predicted to be of African ancestry (3863/sample).

### Genome STRiP CNVDiscovery variant calling and genotyping

Genome STRiP (svtoolkit 2.00.1611) CNVDiscovery^29^, a population level read-depth based caller, was used to identify and genotype biallelic duplications and deletions as well as multiallelic CNVs (mCNVs) with suggested discovery parameters for deeply sequenced genomes (window size: 1000 bp, window overlap: 500 bp, minimum refined length: 500 bp, boundary precision: 100 bp, reference gap length: 1000). Because Genome STRiP is sensitive to differences in cell RR between samples derived from different cell types as well as in sequencing depth, we ran CNVDiscovery separately for iPSCORE fibroblast and blood samples and HipSci fibroblast samples. At the midstage of Genome STRiP discovery, 10 iPSCORE samples and 6 HipSci samples were removed from their respective discovery runs due to excessive variation in the number of calls per sample (exceeding the median call rate across all samples plus three median absolute deviations). To produce a call set where all sites were genotyped in all samples, sites discovered in either iPSCORE or HipSci samples were next genotyped using SVGenotyper in the opposite set (genotyping separately within these respective sets) and the combined list of discovered sites was genotyped in the remaining HipSci iPSC samples, which were excluded from discovery. Using this strategy, the Genome STRiP dataset was not biased by the presence of somatic CNVs in iPSCs, and differences due to WGS library preparation specific to each study were minimized. Additionally, output VCF files from genotyping each subset of samples were annotated to match those from variant discovery using the SVAnnotator (-A CopyNumberClass, \-A CNQuality\-A VariantsPerSample\-A NonVariant\-A Redundancy) to ensure that quality metric information was available for each variant within each subset of samples for downstream processing.

### Genome STRiP CNVDiscovery RR analysis

A commonly suggested filtering parameter for SV detection is the per site quality score GSCNQUAL, described as being comparable for filtering of both duplication and deletion events^60^. We thus tested the RR of Genome STRiP variants ascertained in iPSCORE samples as well as the replication of variants ascertained in the HipSci fibroblast samples (Supplementary Fig. 3a, b). Here we found that GSCNQUAL was highly correlated with RR in both twin pairs and iPSCs, but duplications and mCNVs had higher RR among twin pairs than iPSC–fibroblast pairs. Furthermore, deletions in both iPSCORE and HipSci sites were more reproducible under less stringent filtering than duplications and mCNVs. We selected 2, 12, and 14 as the minimum GSCNQUAL score required for deletions, mCNVs, and duplications, respectively. We then filtered variants that were monoallelic in the dataset as well as sites that had more than 10% of non-iPSC genotypes marked as low quality (LQ format field). These standard filters were applied before proceeding to combine the discovery sets of iPSCORE and HipSci and other data processing.

### Genome STRiP CNVDiscovery variant redundancy collapsing

To collapse redundant variants that were obtained through separate SV discovery for iPSCORE and HipSci samples, we first filtered the HipSci discovery set and the iPSCORE discovery set to those passing filters described above, and then intersected the call sets using bedtools^61,62^. Overlapping sites were required to meet the following criteria in order to be considered redundant: (1) at least 50% reciprocal overlap; (2) Pearson correlation coefficient in the copy numbers of non-iPSC samples > 0.95; and (3) differences in less than 5% of non-mode genotypes in non-iPSC samples. To process these overlaps, we considered cases where two sites exactly overlapped (same coordinates), choosing the site with the largest sum of GSCNQUAL scores from iPSCORE and HipSci (Non-iPSC) samples sets as the high confidence primary site and marking the other as redundant. Pairs of sites with exact overlaps were then removed from the analysis, and the remaining intersections were processed using a graph-based method similar to the one developed for Speedseq. Briefly, overlapping sites (nodes) were connected by edges weighted according to the average percentage overlap (the average of the percentage overlap of site B with A and the percentage overlap of site A with B) and which variant had the largest sum of GSCNQUAL scores from iPSCORE and HipSci (non-iPSC) samples. Then, we iterated through connected components of the graph; chose the pair of sites that had the highest average overlap; and marked the variant with the largest sum of GSCNQUAL scores as the primary site and the other variants in the cluster as redundant. For the X chromosome, the computation of correlation and differences among non-mode samples was done separately for males and females, requiring that sites pass criteria in males, females, or both males and females, depending on whether each subgroup had variability. This was done to control for bias in correlation coefficients due to the difference in reference copy number for males and females on the X chromosome. Overall, this process resulted in 12,757 sites being reduced to 6341 non-redundant primary sites.

### Genome STRiP CNVDiscovery stitching of CNVs

Genome STRiP occasionally reports a single CNV as several adjacent CNVs^19^. To address this issue, we analyzed sites that passed filtering, and were non-redundant, computing the correlation and distance between every pair of adjacent sites. We observed high genotype correlation between sites that overlapped or were close to each other (within ~40 kb) (Supplementary Fig. 4a). Pairs of sites were considered for stitching into a single CNV if they had high overall correlation (*r* > 0.9) between copy number genotypes and at least 80% concordance between copy number genotypes of *non-mode* samples for each variant (union). Because variants that are very far from one another are less likely to be fragmented variant calls, we also selected a maximum distance between a pair of variants to consider for stitching. To do so, we examined the number and percentage of adjacent variant pairs that passed genotype correlation requirements at different distance thresholds, and selected 30 kb, which maximized the number and percentage of pairs passing these requirements (Supplementary Fig. 4b). We then identified correlated adjacent CNVs to be stitched using a graph-based method: (1) a genotype correlation matrix was created for all the CNVs on each chromosome using estimated copy numbers across samples; (2) a graph was drawn with CNVs as nodes, connecting a pair of CNVs with an edge if they resided on the same chromosome and had correlation from their copy number estimates >0.9; (3) for each connected component in the graph with more than a single CNV, CNVs were sorted by position and each adjacent pair was examined for potential stitching; and (4) CNVs were merged if they passed the correlation/concordance criteria described above and were within 30 kb of one another. This approach ensured that only highly correlated adjacent CNVs were merged. In cases where a set of CNVs was chosen to be stitched, a new breakpoint spanning the start point of the first CNV to the end point of the last CNV (sorted by start point) was defined, referred to hereafter as the stitch breakpoint, while the other CNVs in the cluster were considered constituent sites. Note that in cases when a stitch cluster was made up of a single CNV containing one or more smaller CNVs, the large CNV was identified as a stitch breakpoint. Overall, this process lead to 3558 sites being combined into 1252 putative stitch breakpoints, 355 of which were large breakpoints in the call set that contained smaller breakpoints, and 897 were new breakpoints. The set of 897 new stitch breakpoints (not already genotyped in our set) were then genotyped across all samples using Genome STRiP SVGenotyper (CNVDiscovery), separately for iPSCORE samples, HipSci fibroblast samples, and HipSci iPSC samples (as was described in initial discovery 3.3.1). Finally, we compared the genotypes of the stitched breakpoint with the genotypes of the constituent sites, and those that did not have high correlation (average *r* < 0.9 across all constituents) were unstitched, and if the stitch breakpoint was one of the 897 new breakpoints genotyped, it was marked for filtering. If the new stitched breakpoint had over 10% low-quality flagged genotypes (LQ) or was non polymorphic, the stitch cluster was also unstitched, and the breakpoint marked for filtering.

The vast majority of new stitch breakpoints were closely correlated with the constituents (862/897, 96%), suggesting that our stitching strategy indeed identified single CNVs that were broken into fragments (Supplementary Fig. 4c). An additional 7/862 correlated sites failed low-quality genotype filtering criteria, yielding 855/897 (95%) new stitch breakpoints which passed all criteria. Overall, the process yielded 1207 unique sites (855 newly stitched sites and 353 sites that had been previously genotyped) comprising 2–30 distinct CNVs each (Supplementary Fig. 4d). For analysis of the non-redundant set, we filtered these constituent sites and retained the stitch breakpoints. After the filtering, deduplication, and stitching process, remaining non-redundant variants had high replication fractions in each individual twin pair and fibroblast iSPC pair (Supplementary Fig. 3c) and high average RRs on a per site basis (Fig. 3a).

### Genome STRiP CNVDiscovery batch effect and Hardy Weinberg

After filtering, variant collapsing and stitching, we tested for differences in allele distribution and deviations from HWE as described above (see Batch effects and Hardy–Weinberg equilibrium filtering). Non-mode allele frequency was highly correlated between unrelated samples from iPSCORE and HipSci (Supplementary Fig. 3d) though a small number of variants (276/10,302 autosomal CNVs) were identified as having possible differences in allele distribution or deviation from HWE.

### Genome STRiP CNVDiscovery calls per sample

After variant calling and collapsing, we observed approximately the same number of calls per sample among iPSCORE and HipSci fibroblast samples, and no notable outliers among them (Supplementary Fig. 3e). As with other variant callers, we saw larger numbers of calls per sample among samples from the African predicted superpopulation (~28% more calls per sample). Additionally, we found a small number of low-quality genotypes per sample (Supplementary Fig. 3f) on the samples from which we performed discovery. HipSci iPSCs have higher rates of low-quality genotypes because they were excluded from filtering that of sites based on their percentage of genotypes that were tagged as low-quality (FORMAT = LQ) because they were genotyped separately and excluded from the CNVDiscovery pipeline. These results suggest that the discovery and genotyping approach was successful in preventing systematic batch effect variants.

### Genome STRiP LCNVDiscovery variant calling

To identify CNVs longer than 100 kb, which we refer to as long CNVs (LCNVs) we used the LCNVDiscovery module of the Genome STRiP toolkit (svtoolkit 2.00.1611). This pipeline uses information from depth of coverage in fixed-size bins across the genome, and while sample normalization is performed across samples, individual samples are called separately. Prior to LCNVDiscovery, we generated depth profiles for all genomes using GenerateDepthProfiles with suggested parameters (maximumReferenceGapLength = 1000, profileBinSize = 10,000). Then, similar to our approach in Genome STRiP CNVDiscovery, iPSCORE samples, Hipsci fibroblasts, and HipSci iPSCs were processed separately when running the LCNVDiscovery module (maxDepth = 50). We collected the calls from each sample and filtered them with the suggested parameters (NBINS ≥ 10 and a SCORE ≥ 1000). Sites that were entirely contained within the centromere or overlapped the entire centromere were removed and variant sites were required to have an absolute copy number greater than 2.75 or less than 1.25 for duplications and deletions, respectively (Supplementary Fig. 5a).

### Genome STRiP LCNVDiscovery variant processing and QC

Genome STRiP LCNVDiscovery identifies sites per individual sample, so it is necessary to identify redundant sites that are called in different samples. To find redundant CNVs representing a single breakpoint, sites with a reciprocal overlap of at least 80% were grouped into clusters and a single breakpoint spanning the minimum start position to the maximum end position of CNVs in the group was used to represent the merged site. Individual CNVs that were within these clusters were marked as merged constituents, and excluded from non-redundant set, while those that did not overlap with CNVs from another individual were considered unique variants that were present in only a single sample (Supplementary Fig. 5a, b). Absolute copy number estimates were rounded in order to produce integer copy number estimates similar to Genome STRiP CNVDiscovery. We identified 73 redundant sites comprising 2–19 CNVs detected in individuals. On average, twin RRs of the filtered variants was >75% but very few large common variants were identified (Supplementary Fig. 5c). After filtering and collapsing variants, we obtained 432 unique LCNV sites, with 200 duplications, 166 deletions, and 66 mCNV (size range: 100 kb to 5 Mb, Supplementary Fig. 5d). On average each individual had four large duplications and three large deletions (Supplementary Fig. 5e).

### MELT variant calling

MEIs were called using the Mobile Element Locator Tool (MELT)^47^. We used the MELT (v2.0.2) SPLIT workflow to discover, genotype, merge, and annotate MEI calls for ALU, SVA, and LINE1 elements. We also included discovered 1KGP MEI sites^18^ as priors in MELT GroupAnalysis.

### MELT RR analysis and filter selection

While MELT does not output quantitative quality scores, it does flag variants that meet one or more of several criteria. These criteria include: (1) sites that overlap low-complexity regions (lc), (2) have more than 25% missing genotypes (s25), (3) have a ratio of evidence for the left and right breakpoint (LP/RP) that is >2 standard deviations from the ratio among all other sites (rSD), or (4) have a larger than expected number of discordant read pairs that are also split reads (hDP). We tested whether the flags, or combinations or flags, were associated with RR and found that filtering on all suggested criteria improved RR considerably for detected MEIs, raising it from below 0.6 to ~0.9 for ALU, LINE, and SVA elements (Supplementary Fig. 6a, b). Among these quality metrics, filtering on low complexity resulted in the best improvement compared with the other individual filters; however, filtering on all quality tags was necessary to improve RR to 0.9. Additionally, MELT outputs a quality tranche score from 1 to 5 (defined as ASSESS) that describes the types of evidence used to determine the location of the insertion site. For example, the highest quality insertion sites are given a score of 5, and has a target site duplication sequence flanking the MEI supported by split reads. Filtering with higher quality tranche score thresholds also improved RR, either before or after filtering using all flags. We chose to filter variants that that were flagged for any criteria, and also required a quality tranche score of 5, for maximum stringency and best RR improvement. After filtering, individual twin and fibroblast–iPSC pairs had high replication percentages (>0.9; Supplementary Fig. 6c).

### MELT batch effect and Hardy–Weinberg analysis

We tested all MELT variants for differences in allele distribution and deviation from HWE as described above (see Batch effects and Hardy–Weinberg equilibrium filtering) and found that only 527/9,566 autosomal MEIs had differences in allele distribution (49/527) or showed deviation from HWE (492/527) (Supplementary Fig. 6d). Additionally, non-reference allele frequency in iPSCORE and HipSci was highly correlated (*r* > 0.9), suggesting batch effects did not influence MEI calls.

### MELT calls per sample

MELT variants were highly consistent in calls per sample in both studies (mean 1107 and 1097 calls/sample in iPSCORE and HipSci fibroblast samples, respectively) and in all cell types, while having very few missing genotypes (median 1/sample, Supplementary Fig. 6e, f). We observed an increased number of ALU, LINE1, and SVA elements per sample in samples from individuals of African ancestry (1144 ALU/118 LINE1/53 SVA per sample versus 952 ALU/105 SVA/ 45 SVA sample for Non-African samples from iPSCORE).

### HipSTR variant calling

STR variants were genotyped using the HipSTR algorithm (v0.5.61)^38^ on a set of 1,527,077 GRCh37 autosomal STR regions that were provided by the tool (GRCh37.hipstr_reference.bed.gz). Because only HipSci WGS data were PCR-free, special considerations were required to run HipSTR, as it uses PCR stuttering models to genotype repeats and assumes all WGS samples were generated using the same pipeline. For STR genotyping, PCR-free data produce more accurate genotypes, thus we first ran HipSTR at STR sites in all 446 HipSci samples under standard settings. Next, we genotyped the iPSCORE samples using the HipSci genotypes as references (--ref option). Finally, we genotyped iPSCORE samples separately without using the HipSci genotypes as reference alleles. We used only the diploid genotype option, as we lacked phased SNVs for all samples.

### HipSTR filtering and preliminary RR analysis

To filter HipSTR variants, we first used the supplied filter_vcf.py script with recommended thresholds for individual genotypes (min-call-qual = 0.9, max-call-flank-indel = 0.15, max-call-stutter = 0.15, --min-call-allele-bias = −2, min-call-strand-bias = −2). This procedure converts genotypes that do not pass these thresholds to missing. We examined the number of variant calls per sample and the number of missing genotypes when variants were genotyped in iPSCORE, iPSCORE using HipSci reference alleles, and in HipSci samples (Supplementary Fig. 23). Among iPSCORE samples, we observed a median of 122,249 calls per sample in African ancestry individuals and 111,613 calls per samples in non-African ancestry individuals (Supplementary Fig. 23a–d). While four samples from non-African ancestry individuals had a surprisingly large number of STRs, all but one individual self-reported as having partial African ancestry (Supplementary Fig. 23d). iPSCORE genotypes at HipSci reference alleles had similar numbers of calls per sample (median 110,023/sample) compared to the genotypes from iPSCORE alone (Supplementary Fig. 23e). African ancestry samples, however, had a smaller number of calls using the HipSci reference alleles likely because HipSci did not include African ancestry samples, so the African samples in iPSCORE were only genotyped for STRs discovered in Europeans. HipSci samples had about twice as many calls per sample (222,321 per sample for HipSci fibroblast samples) compared to iPSCORE and fewer missing calls per sample, demonstrating that using PCR-free WGS provides better accuracy for STR genotyping. To obtain a high-quality set of STRs, we required >80% call rate for variants from each subset. We excluded one iPSCORE sample from this missingness calculation that had more than 70,000 missing calls. This filter resulted in high RRs (>92%) in each twin pair for both genotyping methods in iPSCORE, and even higher RRs (>95%) in fibroblast–iPSC pairs for HipSci genotyping likely due to more accurate STR genotyping in the PCR-free WGS (Supplementary Fig. 24). Overall, the RR before all filtering and after processing improved from ~78% to ~94.4% in iPSCORE twins (Fig. 3b).

### Combining the iPSCORE and HipSci data for HipSTR

HipSTR genotypes were combined between iPSCORE and HipSci by creating a single combined VCF file using the HipSci genotypes and iPSCORE genotypes at HipSci alleles. We additionally added iPSCORE genotypes for STRs that were unique to iPSCORE to the VCF file.

### Unifying SpeedSeq and Genome STRiP call sets

Since different variant callers may detect the same variants using different methods, we developed a strategy to integrate variants from Genome STRiP and SpeedSeq call sets that were likely to represent the same site. To approach this problem, we used a graph-based method similar to those used to identify duplicates within SpeedSeq and Genome STRiP prior to this step (see SpeedSeq variant redundancy collapsing and Genome STRiP CNVDiscovery variant redundancy collapsing). To generate clusters of overlapping SVs, we first intersected our filtered Genome STRiP calls (redundant sites removed, GSCNQUAL filtered, stitching sites included, stitched constituents excluded) with filtered SpeedSeq variants (redundant sites removed, standard filters, MSQ filtered) and retained all SV pairs with >50% reciprocal overlap or where one variant completely encompassed a second variant that was at least 40% of the length of the larger variant. SV pairs were required to have the same SV types, with exception being that mCNVs were allowed to match with both duplications and deletions and deletions were allowed to match with rMEI (as they appear as deletions). We built a graph where edges were represented by connected SV pairs that pass these overlap thresholds and SV type compatibility parameters. We iterated through connected components, testing every combination of elements in each connected component, and generating a new graph, connecting pairs of variants if they passed correlation thresholds between copy number genotypes (Genome STRiP) variants or allele balance ratios (SpeedSeq) at the sites. If the connected component contained a duplication and deletion from SpeedSeq and an mCNV from Genome STRiP, SV pairs were allowed to connect if their genotype evidence had a correlation (*R*^2^) > 0, while other components required an *R*^2^ > 0.5. We then iterated through connected components of this new graph and selected the highest degree variant (connected to the most other variants) from each caller with the highest quality score (GSCNQUAL for Genome STRiP, MSQ for SpeedSeq) from which we chose one variant as the primary variant and all other variants as secondary. All variants in each cluster were marked with a cluster ID. In cases where a Genome STRiP deletion overlapped a SpeedSeq rMEI, the SpeedSeq variant was chosen as the primary site, and the Genome STRiP variant was assigned as secondary. In all other scenarios, the Genome STRiP variant was chosen as the primary variant and the SpeedSeq variant was the secondary due to the comparably higher RRs for Genome STRiP variants and the granularity of having integer copy numbers.

This method assures that highly correlated variants with significant overlap are clustered together, and that generally, the larger, higher quality variants are chosen as representative primary sites. Sites that were assigned as primary sites from the intersection clusters, as well as unique variants from either variant call set that were not included in intersection clusters, were then selected to produce a non-redundant set of sites necessary for global analyses of SVs (Figs. 4 and 5).

A range of reciprocal overlap and genotype correlation thresholds were tested when unifying the variant calls (Supplementary Fig. 8). Depending on the stringency of these parameters we found that as many as 14,773 were combined into 6935 (0.1 GT correlation, 0.1 RO) or as few as 9769 were combined into 4875 (0.9 GT correlation, 0.9 RO). Based on this analysis, we chose to combine variants under intermediate parameters of 0.5 GT correlation and 0.5 reciprocal overlap, combining a total of 12,757 variants into 6341 variants (including the sex chromosomes).

### Comparison to SV genotypes from arrays

To estimate the FDR of the merged CNV call set we used 216 MEGA_Consortium_v2 arrays available for iPSCORE samples to perform an intensity rank sum (IRS) test to assess whether the SV genotypes after filtering agree with genotypes from array data. SNP arrays were analyzed using the Illumina GenomeStudio software (v2011.1) and were required to have an overall call rate of <97%. The log(*R* ratio) was obtained from the final report. We used the Genome STRiP Intensity Rank Sum Annotator to compare genotypes for a subset of the SV calls that were present in the 216 samples for which we had array data using the log R ratio as input. Before testing, the intensity matrix was first adjusted for covariates by regressing out the effects of batch and plate on a probe-wise basis using the statsmodels (v0.9.0) linear regression module. To assess our filtering strategy we tested 2563/15,437 SpeedSeq duplications and deletions, and 4233/18,171 Genome STRiP CNVs that were present in at least one of the 216 individuals (before any filtering) and contained at least three probes and computed IRS FDR as in 1KGP^18^. Restricting our analysis to 2376 filtered and deduplicated SpeedSeq variants with array probes, we observed that deletions and duplications had an FDR of 5.35% and 3%, respectively. Similarly, among 1848 filtered and deduplicated Genome STRiP variants containing array probes, we observed that deletions, duplications, and mCNVs had an FDR of 5.4%, 7.8%, and 7%, respectively. These FDR estimates were similar to those in 1KGP and GTEx, although the probe density of arrays limited the number of sites we could test.

### Comparison of i2QTL SVs to 1000 Genomes Project and GTEx SVs

To investigate the quality and completeness of our SV calls, we compared them to GTEx v6p SV calls^19^ which used 147 deeply sequenced whole genomes (median 49.9× depth), and the robustly characterized 1000 Genomes Project Phase-3 call set^18^ derived from 2504 shallowly sequenced samples (7.4× depth). While the GTEx call set contains relatively few samples, the WGS data and variant calling approach were similar to the approach used in i2QTL (Genome STRiP and SpeedSeq), and were thus used as a benchmark. Before analysis, we obtained VCF files with genotypes from 1KGP phase-3 and GTEx V6p (dbGaP accession number phs000424.v7.p1). Phased genotypes from 1KGP SVs were converted to unphased genotypes using the alternative allele information to enable comparison with the unphased SVs from i2QTL and GTEx. This enabled us to compute NMAF for 1KGP and GTEx SVs to match the frequency measures used in this study. Because of the significant diversity of the 1KGP cohort (26 populations, 70% European) as compared to i2QTL (6 subpopulations, 80% European), we filtered the 1KGP data to 1755 European samples, and used variants present in at least one of these samples. For co-discovery analyses, we used non-redundant sites from i2QTL as well as variants that passed filters and were part of redundancy clusters to maximize the potential overlap between sets. To identify putative co-discovered sites between i2QTL and either GTEx or 1KGP, CNVs (DUP, DEL, mCNV), rMEI, and inversions from each call set were intersected using bedtools intersect and co-discovered sites were selected using the following approach: (1) excluding inversions, all variants were required to have at least 25% reciprocal overlap, or if one variant was fully contained within the other, it was required to span at least 20% of the larger variant; inversions were required to have 80% reciprocal overlap; (2) variant classes were required to match with the exception of mCNVs, which were allowed to match with either duplications or deletions; for BND sites, we considered breakpoints within 50 bp of each other to be matching; and (3) because we included 1KGP MEIs as priors in our MELT pipeline, MEIs co-discovered with 1KGP were known, and did not require overlap analysis. For overlap reported with i2QTL, we computed the fraction of sites co-discovered by one or both call sets, considering non-redundant clusters a single site.

### Comparison of i2QTL SVs to gnomAD SVs

We obtained SV calls from 10,738 individuals called from deep WGS (mean 32×) data as part of the gnomAD SV project (v2)^49^. Variants were filtered to those that were present in at least one individual of European ancestry and biallelic duplications, deletions, insertions, and inversions where FILTER was PASS or MULTIALLELIC were retained for comparison to i2QTL (*N* = 135,174). Non-insertion variants were then intersected with i2QTL variants using bedtools intersect and those that had matching variant types and at least 25% reciprocal overlap, or if they were fully contained within one another and were at least 20% of the larger variants length were considered matching. For insertions we used bedtools closest to select breakpoints that were within 50 bp of one another, and those that had less than a twofold difference in length were considered matching (insertional sequence information was unavailable for matching). Note that duplications or deletions were allowed to match with mCNV from either set of variant calls. The proportion of variants matching one another was then measured at different MAF thresholds in each dataset (MAF unrelated in i2QTL and MAF European in gnomAD) (Supplementary Fig. 18).

### Comparison of i2QTL SVs to Genome in a Bottle NA12878

To further investigate the quality and completeness of our SV calls, we sought to compare the performance of our variant calling methods on a Genome in a Bottle benchmark sample (NA12878, HG001) for which SVs had been called previously. To complete this analysis, we first ran variant calling using all 5 algorithms for NA12878 and a subset of 75 iPSCORE samples including 25 unrelated European individuals and 25 pairs of monozygotic twins. Before variant calling, the 300x NA12878 [ftp://ftp-trace.ncbi.nlm.nih.gov/giab/ftp/data/NA12878/NIST_NA12878_HG001_HiSeq_300x] sample was first downsampled to 48x using samtools view -s to obtain similar coverage to iPSCORE samples. After downsampling, reads were realigned using bwa mem to the b37 reference with decoy and sendai virus to match the alignment procedure used to align iPSCORE samples. Variant calling was performed using MELT, SpeedSeq, Genome STRiP (CNVDiscovery), and HipSTR and the downstream calls were processed identically for all tools, with the exception of Genome STRiP. For Genome STRiP, since all genomes were derived from iPSCORE/blood samples, no separate genotyping/discovery was necessary to account for batch effects due to tissue of origin. Variants were filtered under the same parameters as the original variant call set. Benchmark short read and PacBio long read variant calls for NA12878 were obtained from 1KGP^18^ and Mt. Sinai [ftp://ftp-trace.ncbi.nlm.nih.gov/giab/ftp/data/NA12878/NA12878_PacBio_MtSinai]. PacBio SVs were filtered to PASS. Variants were intersected and compared with one another under the same parameters that were used for comparison with gnomAD SV (Supplementary Figs. 19 and 20).

### Wham and Manta variant calling

We used wham (v1.7.0)^25^ to call variants on NA12878 and 75 iPSCORE samples (see Comparison of i2QTL SVs to Genome in a Bottle NA12878) separately for each sample using default parameters. After variant calling, we merged the sites discovered in the individual vcf files using mergeSVcallers (parameters: -s 250 -r 0.5). Using this single merged SV call set, we then genotyped each variant in each individual using SVTyper (v0.1.4) under default parameters.

Additionally, we used manta (v1.6.0)^52^ to call variants on the same set of 76 samples. Variant calling was done separately for each sample using default parameters, and for downstream analysis we used SVs genotyped under the diploid model (diploidSV.vcf.gz). Calls from individual samples were then merged across samples using mergeSVcallers under the same parameters as with wham. Finally, the single set of merged variant sites were then genotyped in each individual using SVTyper (v0.1.4) under default parameters.

Genotyped VCFs for each sample from wham or manta were merged using the vcfpaste.py utility in svtools (0.3.2). Note that this sums the QUAL column across all samples for each site.

### Wham and Manta RR and filter selection

For both manta and wham variant calls we examined the effect of QUAL score filtering on RR (Supplementary Fig. 21). Before any filtering, manta variants had average RRs of 75.4% and 71.7% for deletions and duplications, respectively. For wham variants, the average RR before filtering was 79.5%, 65.7%, and 55.9% for deletions, duplications, and inversions respectively. Filtering variants with increasingly stringent QUAL score was associated with increased RRs (Supplementary Fig. 21a, b); however, a large number of variants were removed even under modest thresholds (Supplementary Fig. 21b, c). We chose to filter all variant classes for both wham and manta by requiring a QUAL score of at least 250, ultimately retaining 4086/28,852 wham variants and 3833/24,799 manta variants. Deletions from wham or manta were highly reproducible at this threshold (>95%) while duplications were slightly less reproducible overall (91% wham, 87.2% manta).

### Unification of wham, manta, Genome STRiP, and SpeedSeq calls

To unify and examine the overlap between variant calling approaches, filtered genome STRiP, SpeedSeq, manta, and wham variants from the NA12878 and 75 iPSCORE sample subset (see Comparison of i2QTL SVs to Genome in a Bottle NA12878) were intersected using bedtools. Calls from each unique pair of calls were intersected, requiring 50% reciprocal overlap and matching variant classes. Finally, we used a graph-based approach to obtain clusters of overlapping variants, connecting each pair of variants that passed overlap criteria with an edge, and then extracting each connected component, which then considered a single site occupying the minimum start position to maximum end position of the variants in the cluster. We then assessed the number of different variant calling approaches supporting each of these variant clusters (Supplementary Fig. 22), and used these merged variant coordinates for further comparison to gnomAD SV.

### SNV and indel calling

For SNV and indel genotype calling we followed the GATK^63^ best practices (version 3.8 accessed June 2018). Unless otherwise mentioned settings for the tools are taken from the best practices or left default. As described above, the HipSci WGS data were aligned to the GRCh37 (ref. ^64^) build of the human reference genome using bwa^56^. After alignment Picard was used to mark duplicates. GATK was used for indel realignment and base-recalibration, and genotypes were called using the GATK haplotype caller in GVCF mode. iPSCORE GVCFs were obtained from dbGAP (phs001325) and were used to perform joint genotyping across all iPSCORE and HipSci samples. We used GATK variant recalibration (TS filter level 99.0) to filter low-quality genotype calls for the called SNVs and indels separately.

### Linkage disequilibrium tagging

For each of the 42,921 non-redundant SVs and STRs that were within 1MB of an expressed gene in iPSCs^53^, we used bcftools^57^ to extract all SNPs 50 kb upstream and downstream. For each SV or STR, we calculated LD as the correlation (Pearson *R*^2^) between the SV/STR genotype and the genotypes of each surrounding SNV or indel in i2QTL Europeans and selected the variant with the strongest LD.

### Reporting summary

Further information on research design is available in the Nature Research Reporting Summary linked to this article.

## Supplementary information

Supplementary Information

Description of Additional Supplementary Information

Supplementary Data 1

Supplementary Data 2

Supplementary Data 3

Reporting Summary

## Data Availability

Variant calls from the main i2QTL dataset for iPSCORE samples are available at dbGaP (phs001325) while these calls for HipSci samples are available from Zenodo (10.5281/zenodo.3835306).

## Code availability

Code used for analyses and variant processing can be found on GitHub [https://github.com/frazer-lab/i2QTL-SV-STR-analysis].

## References

[1] Carvalho CM, Lupski JR (2016). Mechanisms underlying structural variant formation in genomic disorders. Nat. Rev. Genet.

[2] Brandler WM (2018). Paternally inherited cis-regulatory structural variants are associated with autism. Science.

[3] Malhotra D (2011). High frequencies of de novo CNVs in bipolar disorder and schizophrenia. Neuron.

[4] Malhotra D, Sebat J (2012). CNVs: harbingers of a rare variant revolution in psychiatric genetics. Cell.

[5] Michaelson Jacob J (2012). Whole-genome sequencing in autism identifies hot spots for de novo germline mutation. Cell.

[6] Beck M (2015). Craniofacial abnormalities and developmental delay in two families with overlapping 22q12.1 microdeletions involving the MN1 gene. Am. J. Med. Genet. A.

[7] Spielmann M, Klopocki E (2013). CNVs of noncoding cis-regulatory elements in human disease. Curr. Opin. Genet. Dev..

[8] Pearson CE (2003). Slipping while sleeping? Trinucleotide repeat expansions in germ cells. Trends Mol. Med..

[9] Mirkin SM (2007). Expandable DNA repeats and human disease. Nature.

[10] La Spada AR, Taylor JP (2010). Repeat expansion disease: progress and puzzles in disease pathogenesis. Nat. Rev. Genet.

[11] McMurray CT (2010). Mechanisms of trinucleotide repeat instability during human development. Nat. Rev. Genet..

[12] Nelson DL, Orr HT, Warren ST (2013). The unstable repeats–three evolving faces of neurological disease. Neuron.

[13] Spielmann M, Mundlos S (2013). Structural variations, the regulatory landscape of the genome and their alteration in human disease. BioEssays.

[14] Den Dunnen WFA (2017). Trinucleotide repeat disorders. Handb. Clin. Neurol..

[15] Gamazon ER, Nicolae DL, Cox NJ (2011). A study of CNVs as trait-associated polymorphisms and as expression quantitative trait loci. Plos Genet..

[16] Kong SW (2012). Characteristics and predictive value of blood transcriptome signature in males with autism spectrum disorders. PLoS ONE.

[17] Schlattl A, Anders S, Waszak SM, Huber W, Korbel JO (2011). Relating CNVs to transcriptome data at fine resolution: assessment of the effect of variant size, type, and overlap with functional regions. Genome Res..

[18] Sudmant PH (2015). An integrated map of structural variation in 2,504 human genomes. Nature.

[19] Chiang C (2017). The impact of structural variation on human gene expression. Nat. Genet..

[20] Hehir-Kwa JY (2016). A high-quality human reference panel reveals the complexity and distribution of genomic structural variants. Nat. Commun..

[21] Kosugi S (2019). Comprehensive evaluation of structural variation detection algorithms for whole genome sequencing. Genome Biol..

[22] Rausch T (2012). DELLY: structural variant discovery by integrated paired-end and split-read analysis. Bioinformatics.

[23] Fan X, Abbott TE, Larson D, Chen K (2014). BreakDancer: identification of genomic structural variation from paired-end read mapping. Curr. Protoc. Bioinformatics.

[24] Layer RM, Chiang C, Quinlan AR, Hall IM (2014). LUMPY: a probabilistic framework for structural variant discovery. Genome Biol..

[25] Kronenberg ZN (2015). Wham: identifying structural variants of biological consequence. PLoS Comput Biol..

[26] Abyzov A, Urban AE, Snyder M, Gerstein M (2011). CNVnator: an approach to discover, genotype, and characterize typical and atypical CNVs from family and population genome sequencing. Genome Res..

[27] Klambauer G (2012). MOPS: mixture of Poissons for discovering copy number variations in next-generation sequencing data with a low false discovery rate. Nucleic Acids Res..

[28] Zhu M (2012). Using ERDS to infer copy-number variants in high-coverage genomes. Am. J. Hum. Genet..

[29] Handsaker RE (2015). Large multiallelic copy number variations in humans. Nat. Genet..

[30] Lin K, Smit S, Bonnema G, Sanchez-Perez G, de Ridder D (2015). Making the difference: integrating structural variation detection tools. Brief. Bioinformatics.

[31] Becker T (2018). FusorSV: an algorithm for optimally combining data from multiple structural variation detection methods. Genome Biol..

[32] Chaisson MJP (2019). Multi-platform discovery of haplotype-resolved structural variation in human genomes. Nat. Commun..

[33] Chaisson MJP (2015). Resolving the complexity of the human genome using single-molecule sequencing. Nature.

[34] Collins RL (2017). Defining the diverse spectrum of inversions, complex structural variation, and chromothripsis in the morbid human genome. Genome Biol..

[35] Audano PA (2019). Characterizing the major structural variant alleles of the human genome. Cell.

[36] Levy-Sakin M (2019). Genome maps across 26 human populations reveal population-specific patterns of structural variation. Nat. Commun..

[37] Gymrek, M. et al. Abundant contribution of short tandem repeats to gene expression variation in humans. *Nat Genet*. **48**, 22–29, 10.1038/ng.3461 (2016). 10.1038/ng.3461 PMC4909355 26642241

[38] Willems T (2017). Genome-wide profiling of heritable and de novo STR variations. Nat. Methods.

[39] D’Antonio M (2018). Insights into the mutational burden of human induced pluripotent stem cells from an integrative multi-omics approach. Cell Rep..

[40] DeBoever C (2017). Large-scale profiling reveals the influence of genetic variation on gene expression in human induced pluripotent stem cells. Cell Stem Cell.

[41] Panopoulos AD (2017). iPSCORE: a resource of 222 iPSC lines enabling functional characterization of genetic variation across a variety of cell types. Stem Cell Rep..

[42] Kilpinen H (2017). Common genetic variation drives molecular heterogeneity in human iPSCs. Nature.

[43] Streeter I (2017). The human-induced pluripotent stem cell initiative-data resources for cellular genetics. Nucleic Acids Res..

[44] Kilpinen H (2017). Corrigendum: common genetic variation drives molecular heterogeneity in human iPSCs. Nature.

[45] Auton A (2015). A global reference for human genetic variation. Nature.

[46] Chiang C (2015). SpeedSeq: ultra-fast personal genome analysis and interpretation. Nat. Methods.

[47] Gardner EJ (2017). The Mobile Element Locator Tool (MELT): population-scale mobile element discovery and biology. Genome Res..

[48] Ramachandran S (2005). Support from the relationship of genetic and geographic distance in human populations for a serial founder effect originating in Africa. Proc. Natl. Acad. Sci. USA.

[49] Collins, R. L. et al. An open resource of structural variation for medical and population genetics. Preprint at https://www.biorxiv.org/content/10.1101/578674v1 (2019).

[50] Zook JM (2016). Extensive sequencing of seven human genomes to characterize benchmark reference materials. Sci. Data.

[51] Parikh H (2016). svclassify: a method to establish benchmark structural variant calls. BMC Genomics.

[52] Chen X (2016). Manta: rapid detection of structural variants and indels for germline and cancer sequencing applications. Bioinformatics.

[53] Jakubosky, D. et al. Properties of structural variants and short tandem repeats associated with gene expression and complex traits. *Nat Commun*. 10.1038/s41467-020-16482-4 (2020). 10.1038/s41467-020-16482-4 PMC7286898 32522982

[54] Sankar PL, Parker LS (2017). The Precision Medicine Initiative’s All of Us Research Program: an agenda for research on its ethical, legal, and social issues. Genet. Med.

[55] Brown, J., Pirrung, M. & McCue, L. A. FQC Dashboard: integrates FastQC results into a web-based, interactive, and extensible FASTQ quality control tool. *Bioinformatics*, 10.1093/bioinformatics/btx373 (2017). 10.1093/bioinformatics/btx373 PMC5870778 28605449

[56] Li H, Durbin R (2009). Fast and accurate short read alignment with Burrows-Wheeler transform. Bioinformatics.

[57] Li H (2009). The Sequence Alignment/Map format and SAMtools. Bioinformatics.

[58] Tarasov A, Vilella AJ, Cuppen E, Nijman IJ, Prins P (2015). Sambamba: fast processing of NGS alignment formats. Bioinformatics.

[59] Li, H. Toward better understanding of artifacts in variant calling from high-coverage samples. *Bioinformatics (Oxford, England)*, 1–9, 10.1093/bioinformatics/btu356 (2014). 10.1093/bioinformatics/btu356 PMC4271055 24974202

[60] Andersson R (2014). An atlas of active enhancers across human cell types and tissues. Nature.

[61] Quinlan AR (2014). BEDTools: The Swiss-Army Tool for genome feature analysis. Curr. Protoc. Bioinformatics.

[62] Quinlan AR, Hall IM (2010). BEDTools: a flexible suite of utilities for comparing genomic features. Bioinformatics.

[63] McKenna A (2010). The Genome Analysis Toolkit: a MapReduce framework for analyzing next-generation DNA sequencing data. Genome Res..

[64] Church DM (2011). Modernizing reference genome assemblies. PLoS Biol..

