# Supplementary material for: Discovery and quality analysis of a comprehensive set of structural variants and short tandem repeats
Source: Nat Commun. 2020 Jun 10;11:2928. doi: 10.1038/s41467-020-16481-5 (PMC7287045; doi:10.1038/s41467-020-16481-5)
Supplement: Supplementary file 1 — Supplementary Information [file 41467_2020_16481_MOESM1_ESM.pdf]

Supplementary Information

Supplementary Figures

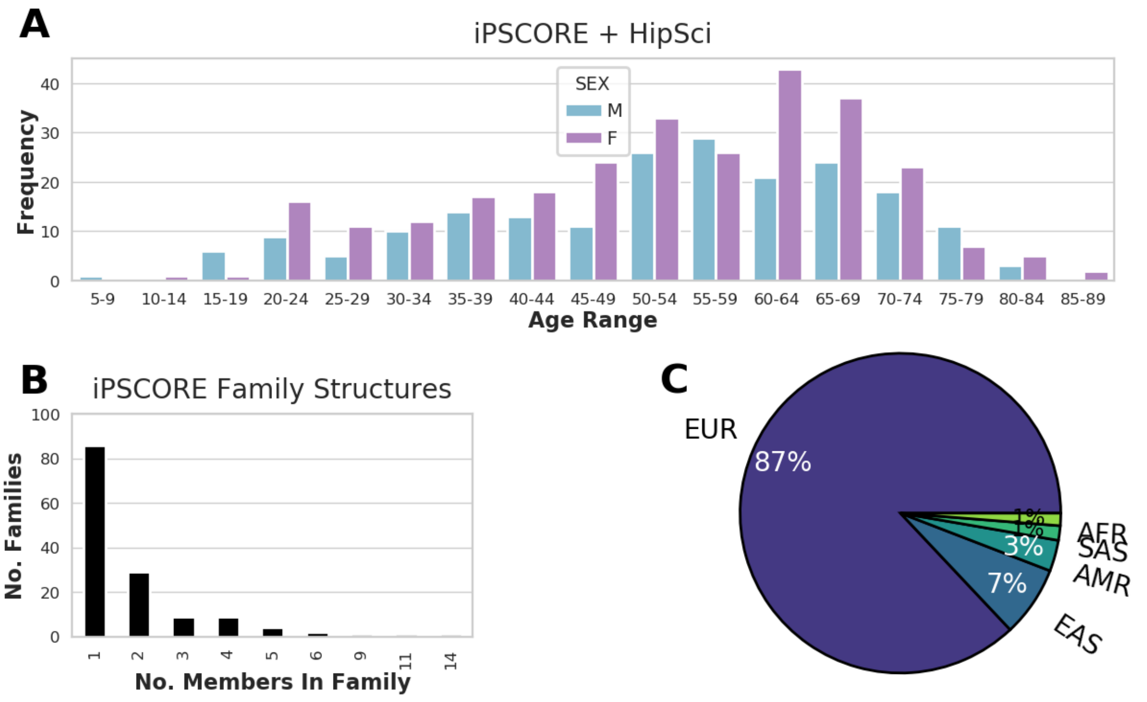

Supplementary Figure 1. i2QTL Subject Information. (A) Age distribution of WGS donors stratified by sex. (B) Size of families in iPSCORE. (C) Number of individuals from iPSCORE and HipSci assigned to each of the 1000 Genomes Project superpopulations using genotype data[1].

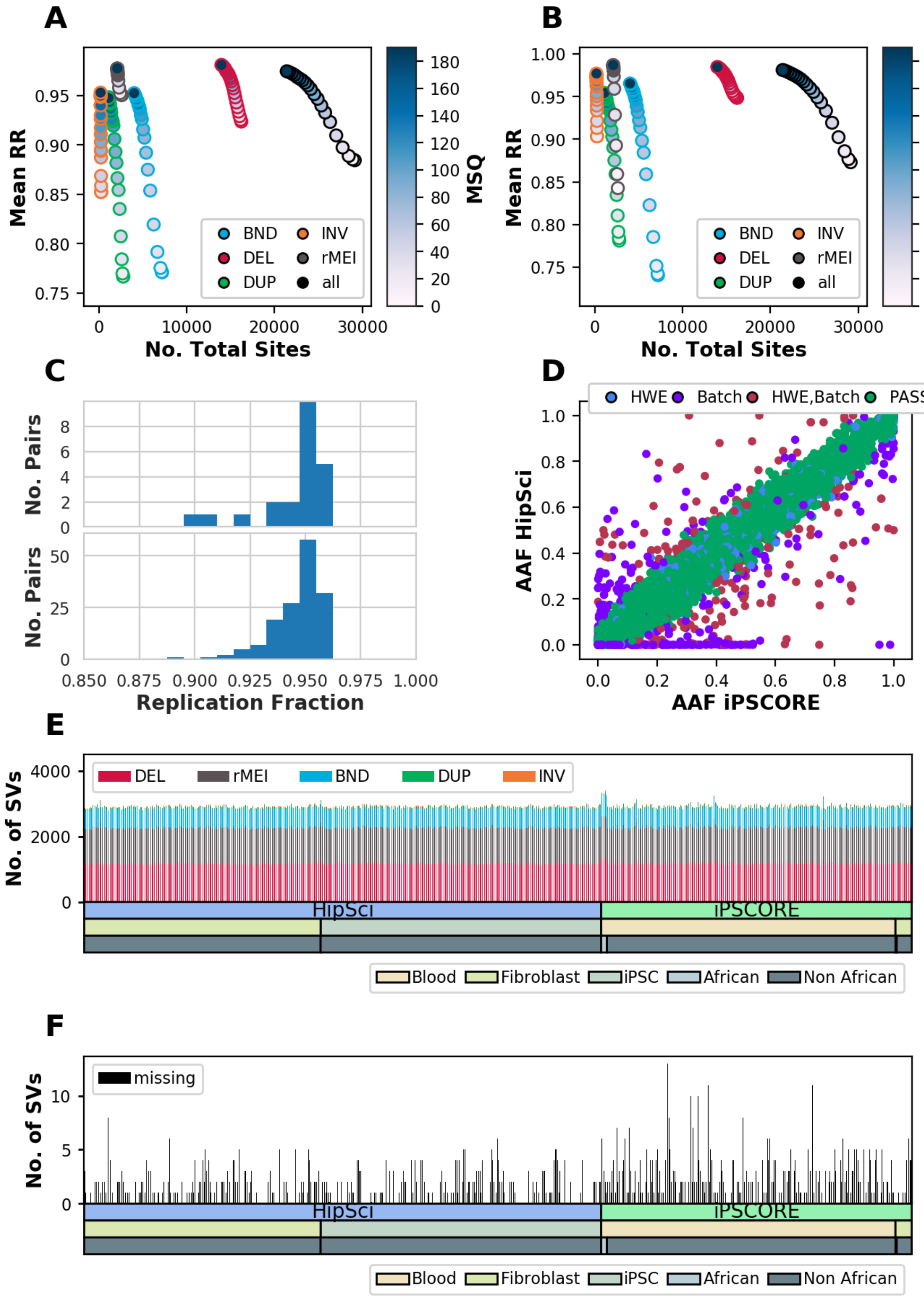
A
B
Mean RR
No. Total Sites
MSQ
BND
DEL
DUP
INV
rMEI
all
C
No. Pairs
Replication Fraction
D
HWE
Batch
HWE,Batch
PASS
AAF HipSci
AAF iPSCORE
E
No. of SVs
DEL
rMEI
BND
DUP
INV
HipSci
iPSCORE
Blood
Fibroblast
iPSC
African
Non African
F
missing

Supplementary Figure 2: SpeedSeq Quality Control (A and B) Replication rate in (A) iPSCORE monozygotic twins and (B) HipSci fibroblast iPSC pairs as a function of the number of total sites that pass filtering thresholds for median sample quality score (MSQ). (C) Replication rate distribution in monozygotic twin pairs (upper) and fibroblast iPSC pairs (lower). (D) Comparison of non-reference allele frequency of calls in iPSCORE unrelated samples and HipSci fibroblast samples, colored by whether their genotype distributions were flagged for deviation from Hardy Weinberg Equilibrium (blue) or potential systematic differences between genotypes in HipSci and genotypes in iPSCORE ("Batch", purple), or both (red). (E) Number of events per individual after filtering for each variant type. (F) Number of events with a missing genotype after filtering.

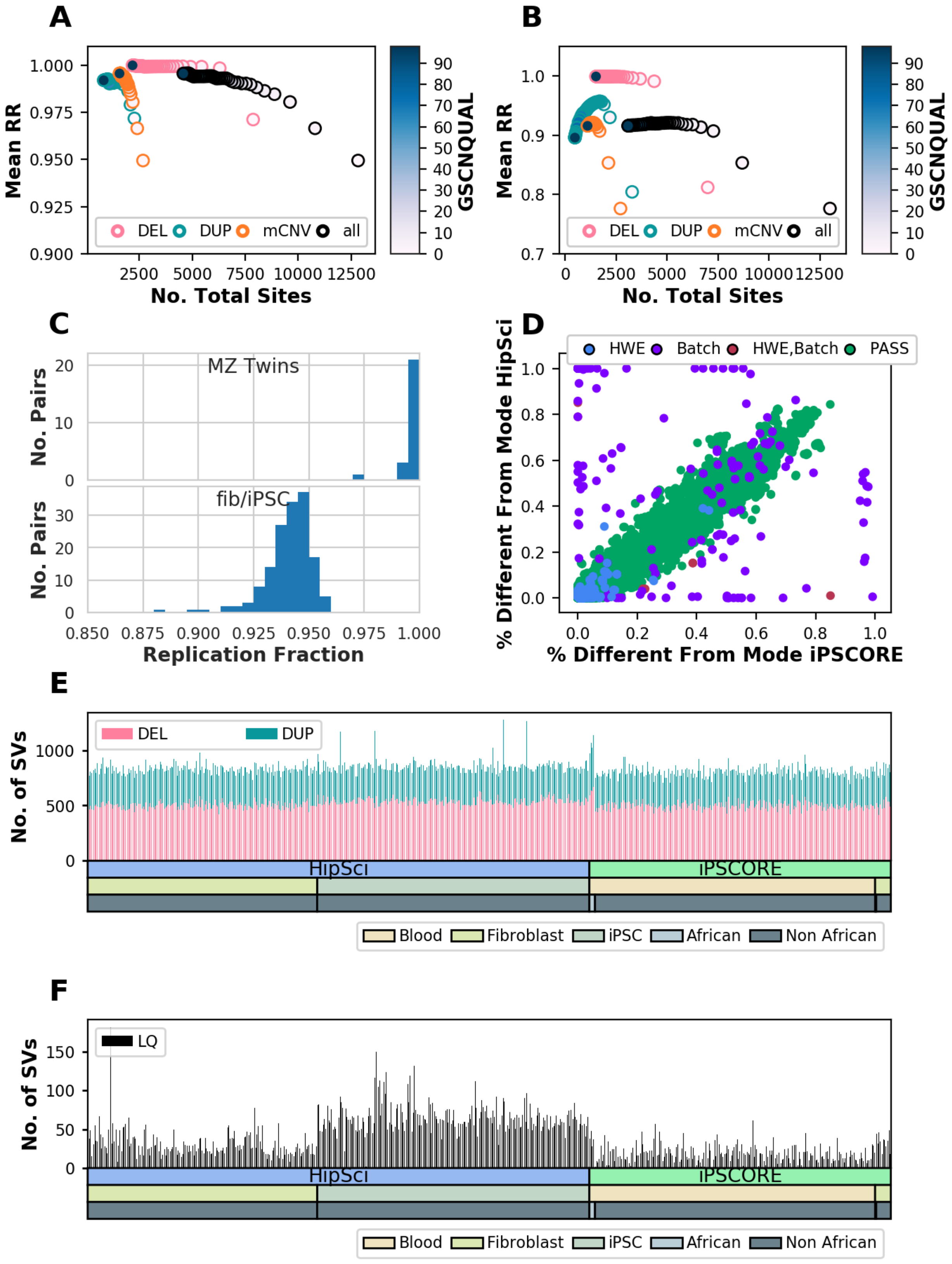
A
B
C
D
E
F
Mean RR
No. Total Sites
GSCNQUAL
DEL
DUP
mCNV
all
MZ Twins
fib/iPSC
No. Pairs
Replication Fraction
HWE
Batch
HWE,Batch
PASS
% Different From Mode HipSci
% Different From Mode iPSCORE
No. of SVs
HipSci
iPSCORE
Blood
Fibroblast
iPSC
African
Non African
LQ

Supplementary Figure 3: Genome STRiP Quality Control. (A, B) Replication rate in (A) iPSCORE monozygotic twins and (B) HipSci fibroblast iPSC pairs as a function of the number of total sites that pass filtering thresholds for GSCNQUAL. (C) Replication rate distribution in monozygotic twin pairs (upper) and fibroblast iPSC pairs (lower). (D) Comparison of percent of samples different from the copy-number mode in iPSCORE unrelated samples and HipSci fibroblast samples, colored by whether their genotype distributions were flagged for deviation from Hardy Weinberg Equilibrium (blue) or potential systematic differences between genotypes in HipSci and genotypes in iPSCORE ("Batch", purple), or both (red). (E) Number of duplication or deletion events per individual after filtering. (F) Number of events with a genotype tagged as LQ (low quality) after filtering. Note that we filtered to variants that were less than 10% LQ rate among iPSCORE samples and HipSci fibroblast samples. Therefore, we observed more LQ sites among HipSci iPSCs.

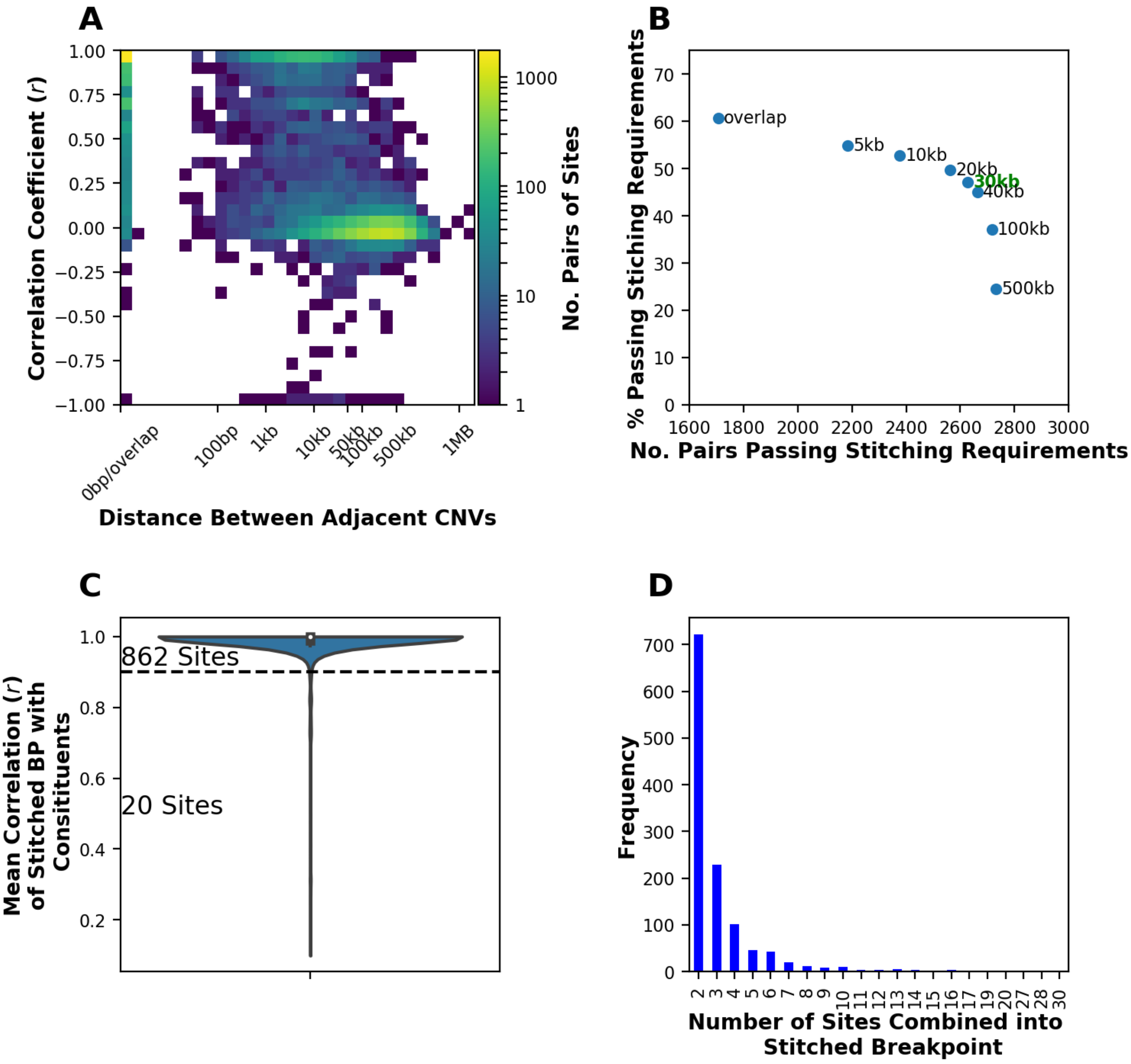

Supplementary Figure 4: Genome STRiP Variant Stitching. (A) 2D histogram showing the distance and correlation between pairs of adjacent CNVs, colored by the number of pairs of sites. This information was used to decide the maximum distance threshold used to stitch neighboring sites. (B) Number of pairs that passed stitching requirements versus the percentage which passed stitching requirements for different thresholds of maximum distance between variants (see Methods). We chose a

threshold of 30kb (green) as this maximized the percent of pairs that passed stitching requirements, and few pairs of sites greater than 30kb apart were correlated. (C) Mean correlation of the stitched breakpoint genotypes with the genotypes of the constituent sites. In cases where the stitched breakpoint correlated at less than 0.9, it was discarded and the site was “unstitched”. Stitch breakpoints were also unstitched if more than 10% of samples had low quality (LQ) genotypes among the 477 iPSCORE and HipSci (fibroblast) samples (D) Number of sites combined into stitched breakpoints.

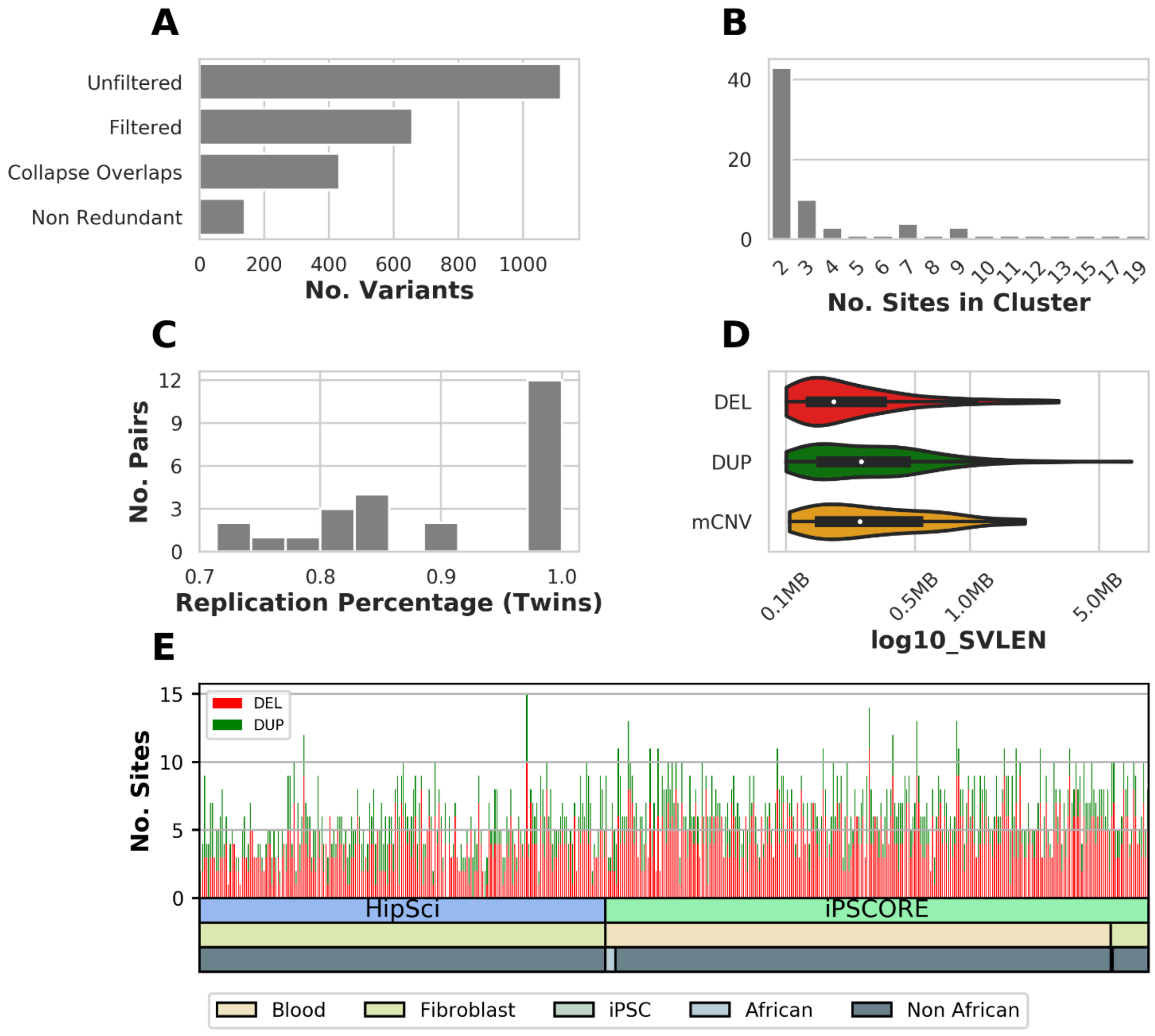

Supplementary Figure 5. Genome STRiP LCNV Quality Control and Filtering. (A) Number of variants identified by the Genome STRiP LCNV pipeline at each filtering step. (B) Because GS LCNV variants are detected separately for each sample, all breakpoints are not genotyped in each sample, and large common variants may have different coordinates in distinct samples. Therefore, we collapsed all variants with reciprocal overlap >80% into single sites. Here we show the number of clusters of overlapping variants and the number of sites per cluster. (C) Replication percentage in each iPSCORE twin pair (D) Length distribution and (E) number of duplications and deletions per sample for variants after filtering and collapsing. Boxplots are contained

within violinplots, and the minimum box edge indicates the first quartile while the maximum box edge indicates the third quartile. White dots in the boxes indicate the median value. Whiskers of the box plot are drawn at the maximum point (upper whisker) or minimum point (lower whisker) that is within 1.5 times the interquartile range (quartile three - quartile one). Points beyond this range are considered outliers (and not plotted) but maximum and minimum values are shown with the range of the outer violinplot.

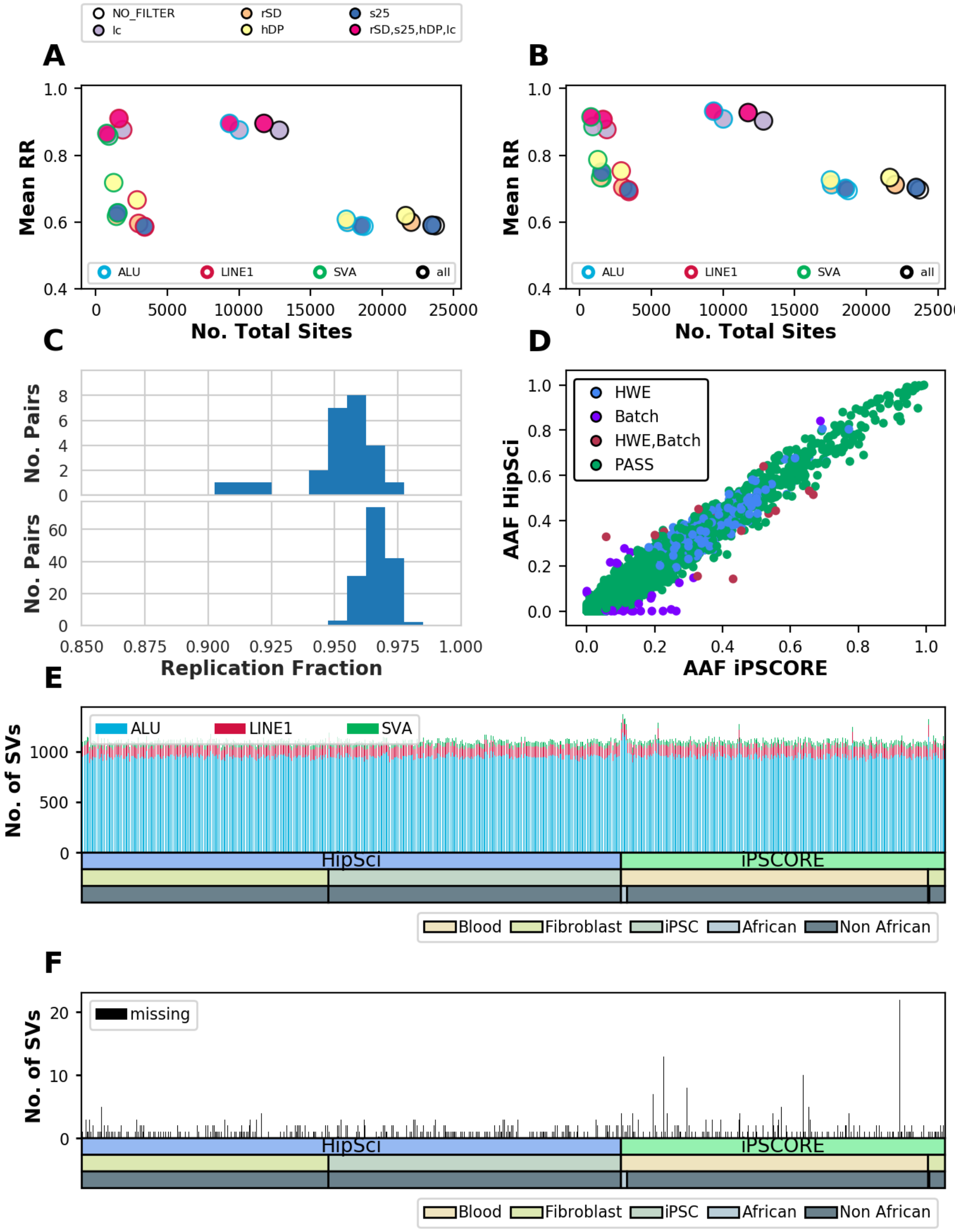
NO_FILTER
lc
rSD
hDP
s25
rSD,s25,hDP,lc
A
B
C
D
E
F
Mean RR
No. Total Sites
ALU
LINE1
SVA
all
No. Pairs
Replication Fraction
HWE
Batch
HWE,Batch
PASS
AAF HipSci
AAF iPSCORE
No. of SVs
HipSci
iPSCORE
Blood
Fibroblast
iPSC
African
Non African
missing

Supplementary Figure 6: MELT Quality Control. (A and B) Replication rate in (A) iPSCORE monozygotic twins and (B) HipSci fibroblast iPSC pairs as a function of the number of total sites that pass filtering thresholds various parameters that are suggested by MELT, colors show how many of these filters were applied. (C) Replication rate distribution in monozygotic twin pairs (upper) and fibroblast iPSC pairs (lower). (D) Comparison of non-reference allele frequency of calls in iPSCORE unrelated samples and HipSci fibroblast samples, colored by whether their genotype distributions were flagged for deviation from Hardy Weinberg Equilibrium (blue) or potential systematic differences between genotypes in HipSci and genotypes in iPSCORE ("Batch", purple), or both (red). (E) Number of events per individual after filtering for each variant type. (F) Number of events with a missing genotype after filtering.

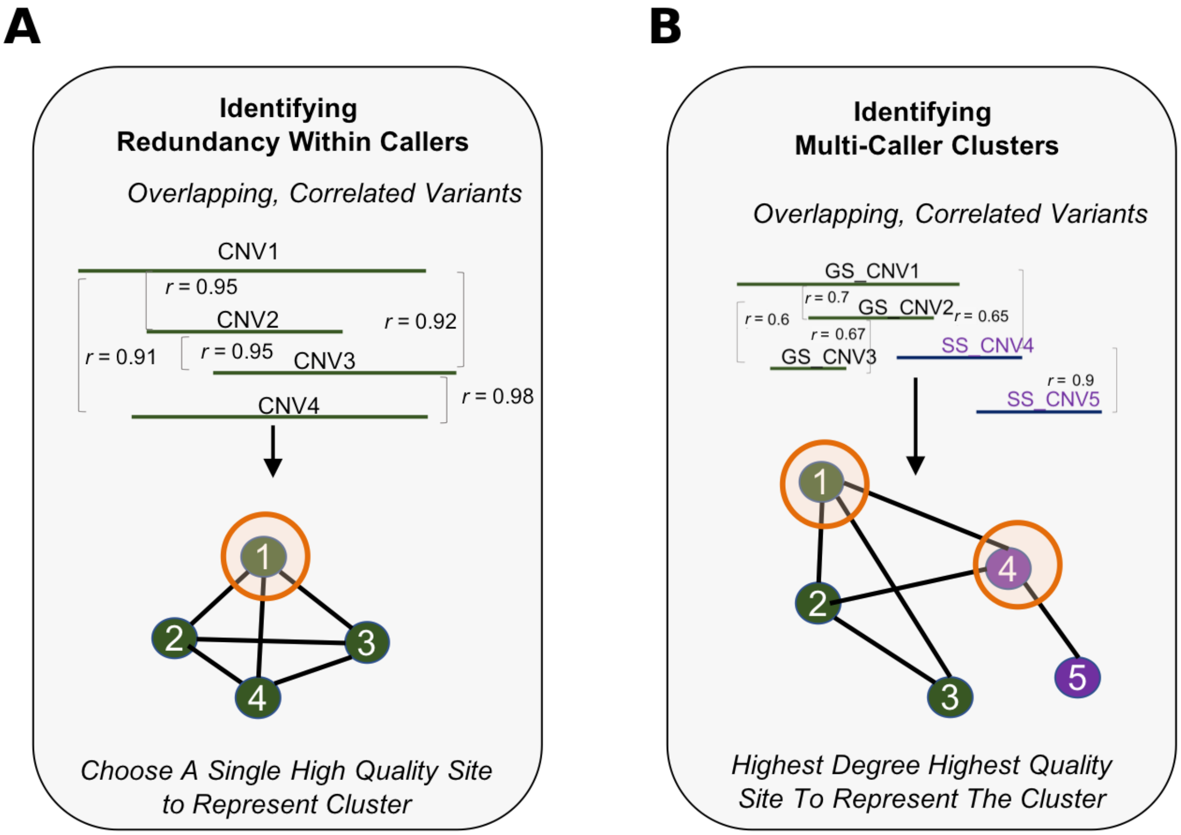

Supplementary Figure 7. Collapsing Redundancies Within and Between Variant Callers. (A) Strategy for collapsing redundancies between callers by identifying clusters of overlapping variants with high correlation between variants. While the approach for SpeedSeq and Genome STRiP was slightly different (Methods), overall, the goal was to select a single high-quality site to represent a cluster of correlated variants. (B) Illustration of the strategy for identifying redundant sites called by multiple algorithms. Here, similar to collapsing variants within caller, overlapping variants from different algorithms that had correlated genotyping information were represented as a graph. Edges were drawn between variants that did not overlap if genotype correlation was above a specific threshold (Methods), and the highest degree, highest quality variant was chosen from either Genome STRiP or SpeedSeq to represent the cluster. In both A and B, "*r*" values between brackets represent pearson correlation coefficients between two variants connected by brackets. Both of these schematics are meant to illustrate the logic of the algorithms to collapse variants, and do not come from the real data.

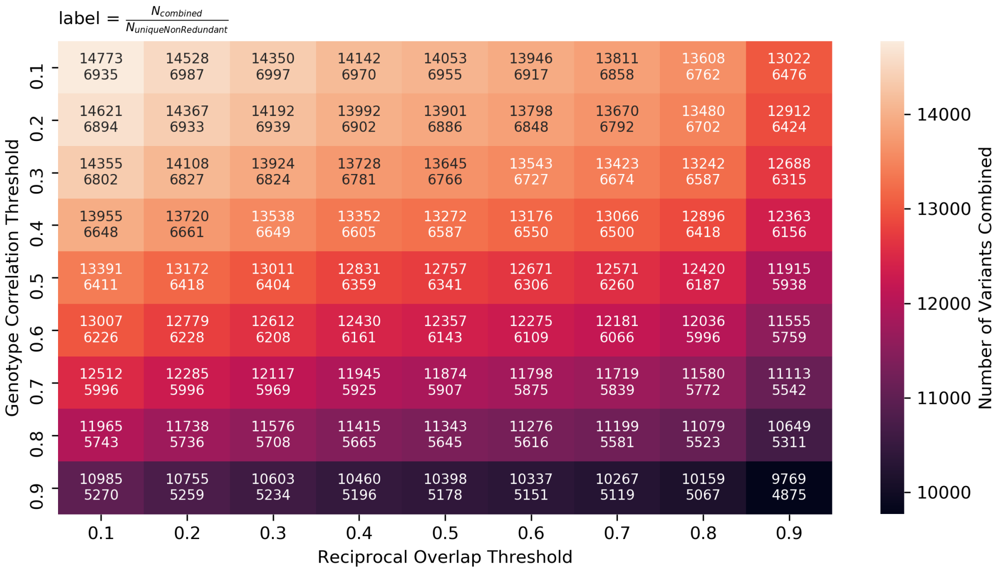

Supplementary Figure 8. Number of Variants Collapsed under Different Overlap and Correlation Parameters. Heatmap showing the performance of between caller redundancy collapsing for each combination of reciprocal overlap threshold (x-axis) and genotype correlation threshold (y-axis). In each cell of the heatmap, we show the total number of SpeedSeq and Genome STRiP variants combined (top number in square) and how many unique variants these represented (bottom number in square). For our final call set we used 0.5 for both genotype correlation threshold and reciprocal overlap threshold.

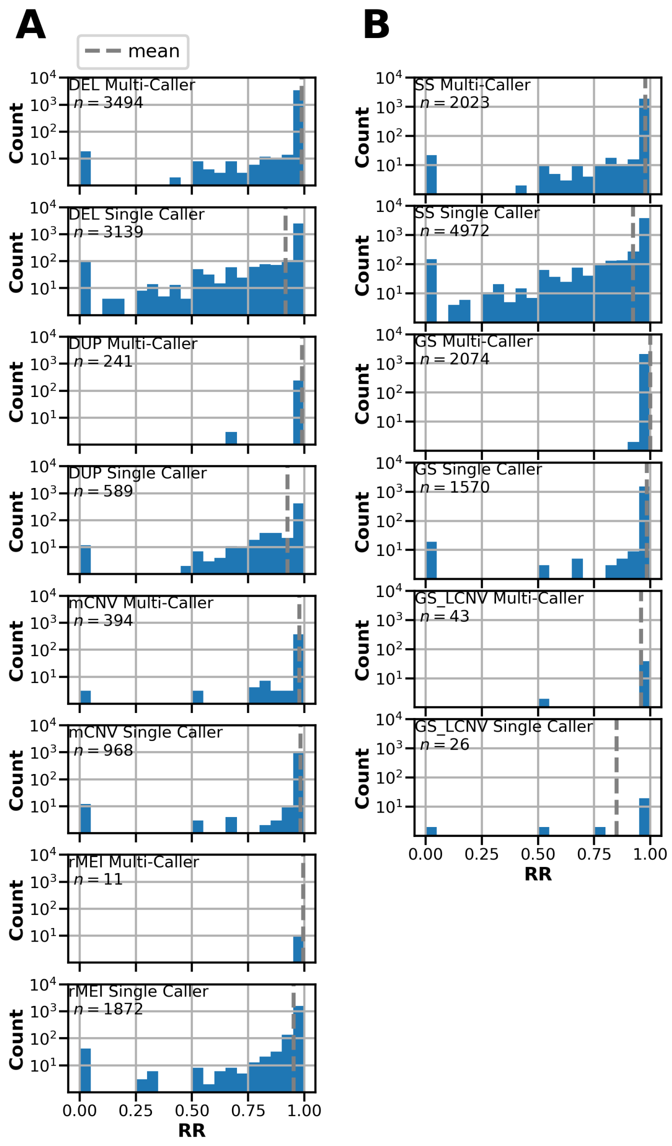

Supplementary Figure 9. Replication Rate of Variants that are Identified by Multiple Callers. (A, B) Average replication rate of variants that were or were not discovered by multiple callers stratified by (A) variant type or (B) variant caller. Error bars indicate 95% confidence interval around the mean.

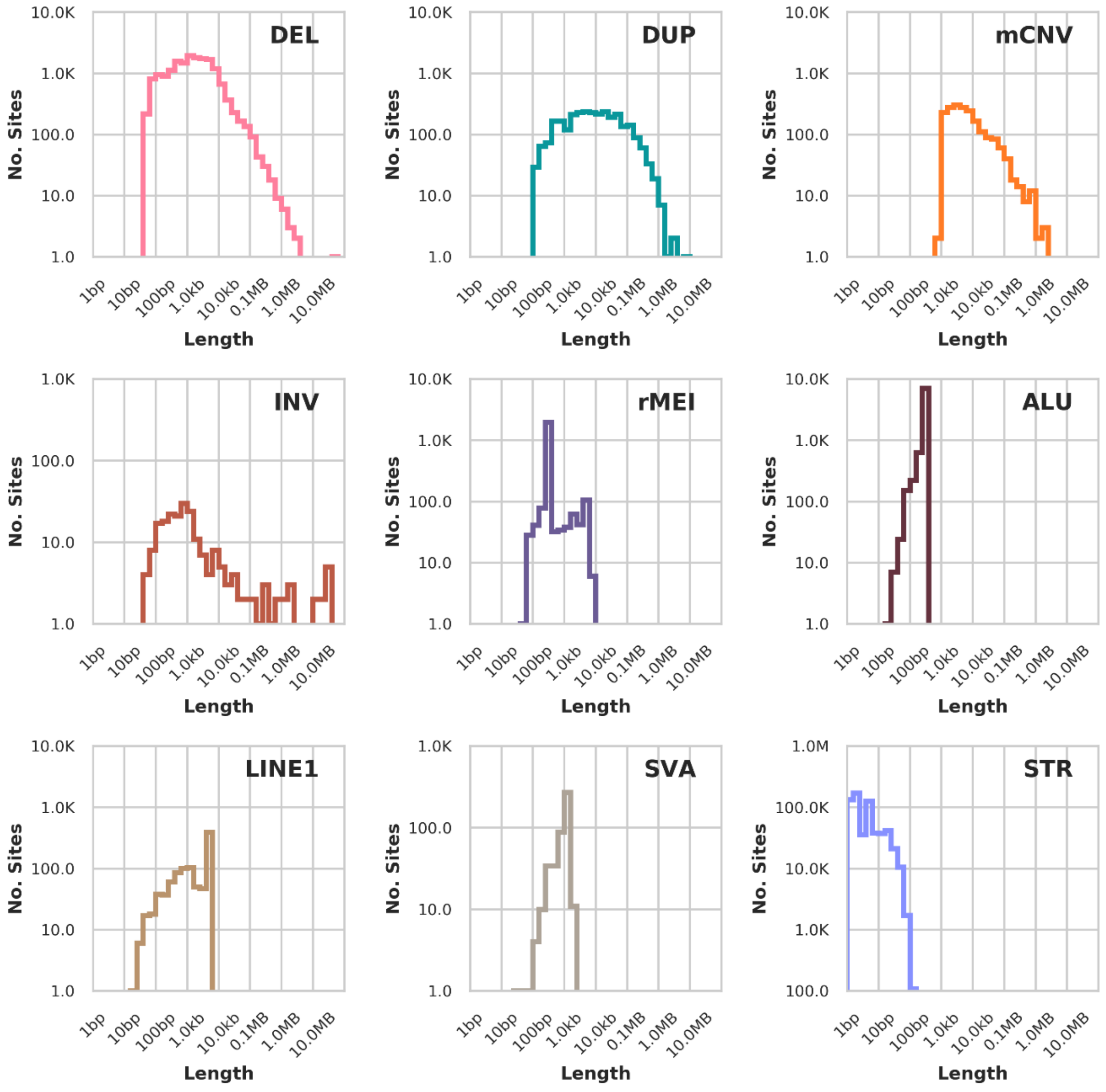

Supplementary Figure 10. Length Distribution of Non-Redundant Variants. Length distribution of variants in the non-redundant call set as identified in 477 individuals. The length for STRs is calculated as the maximum absolute difference in base pairs from the reference allele at a site while the length for CNVs (DEL, DUP, mCNV) is the size of a single copy unit. For rMEI, ALU, LINE1, and SVA the length represents the estimated

insertion size of the variant. For inversions, the length is the distance between the two breakpoints.

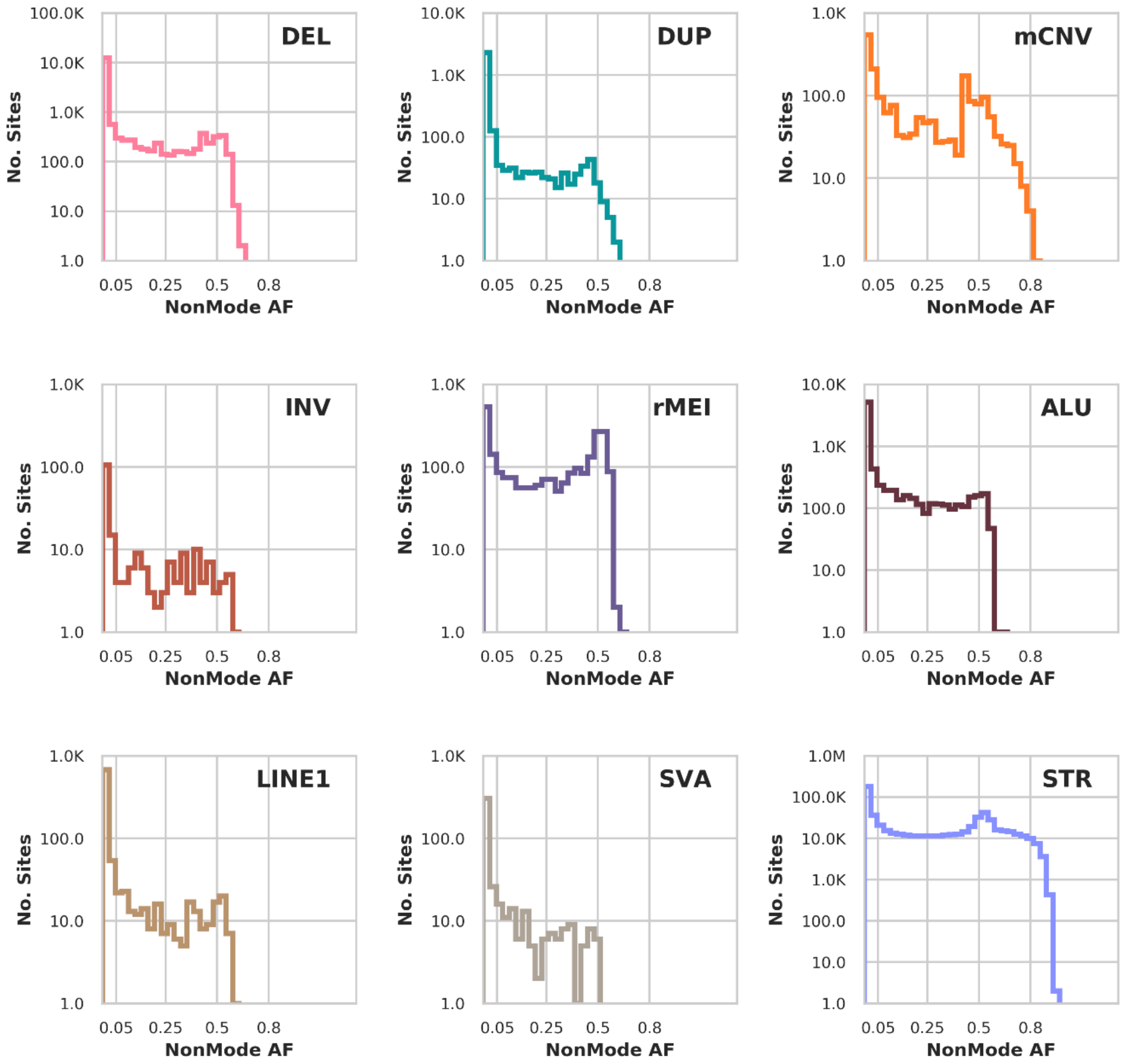

Supplementary Figure 11. Allele Frequency Distribution of Non-Redundant Variants. Distribution of the non-mode allele frequency in each variant class for i2QTL unrelated samples.

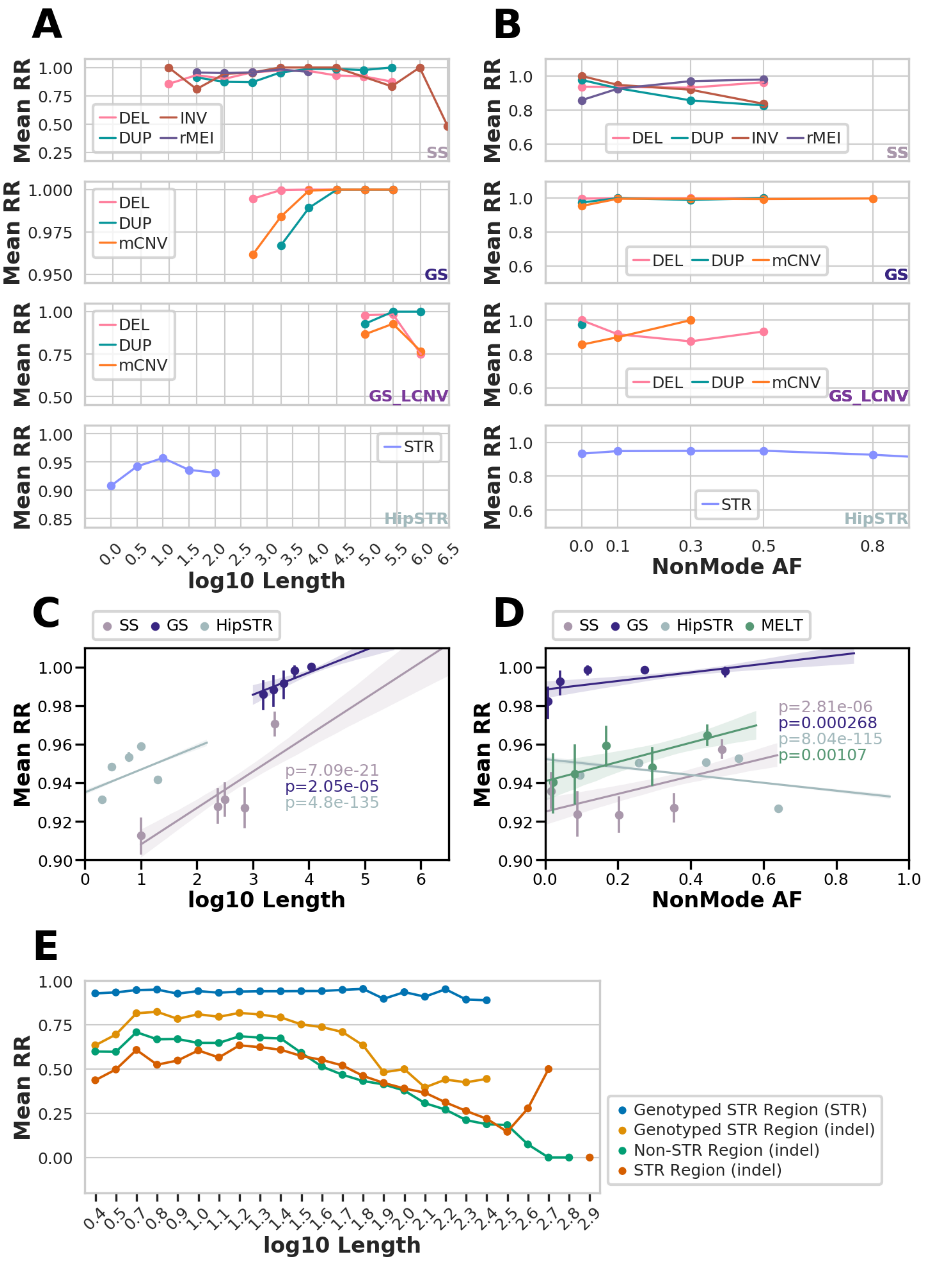

Supplementary Figure 12. Effect of Length and Allele Frequency on Replication Rate Within Callers. (A and B) Replication rate versus log10 variant length (A) and non-mode allele frequency (B) for deduplicated variants passing filters from each variant caller, stratified by variant class, prior to unifying between variant callers. (C and D) Replication rate versus log10 length for each variant caller after regressing out the effect of non-mode allele frequency (C) and versus non-mode allele frequency (D) after regressing out the effect of log10 length. Points represent the centers of equally sized

bins with error bars showing 95% confidence intervals around the mean. Regression lines are shown with shading representing 95% confidence intervals. Regression p values matching the color of their corresponding category are shown, as computed by linear regression, modeling replication rate as a function of nonmode allele frequency and log10 transformed variant length as covariates.(E) Replication rate of indels that overlap STR reference regions that are genotyped as non-reference in at least one individual by HipSTR (orange), or are not polymorphic in HipSTR (red), or do not overlap an STR region (green) divided into bins by length. Each point represents the center of a bin. For A-D, variants were subsetted to those in each caller that passed deduplication within variant caller, segregated in at least one twin pair, and were present in at least one unrelated individual. For these plots, the total number of variants for each variant caller are: GS (n = 3,644), SS (n = 8,934), HipSTR (n = 213,005), MELT (n= 3,601).

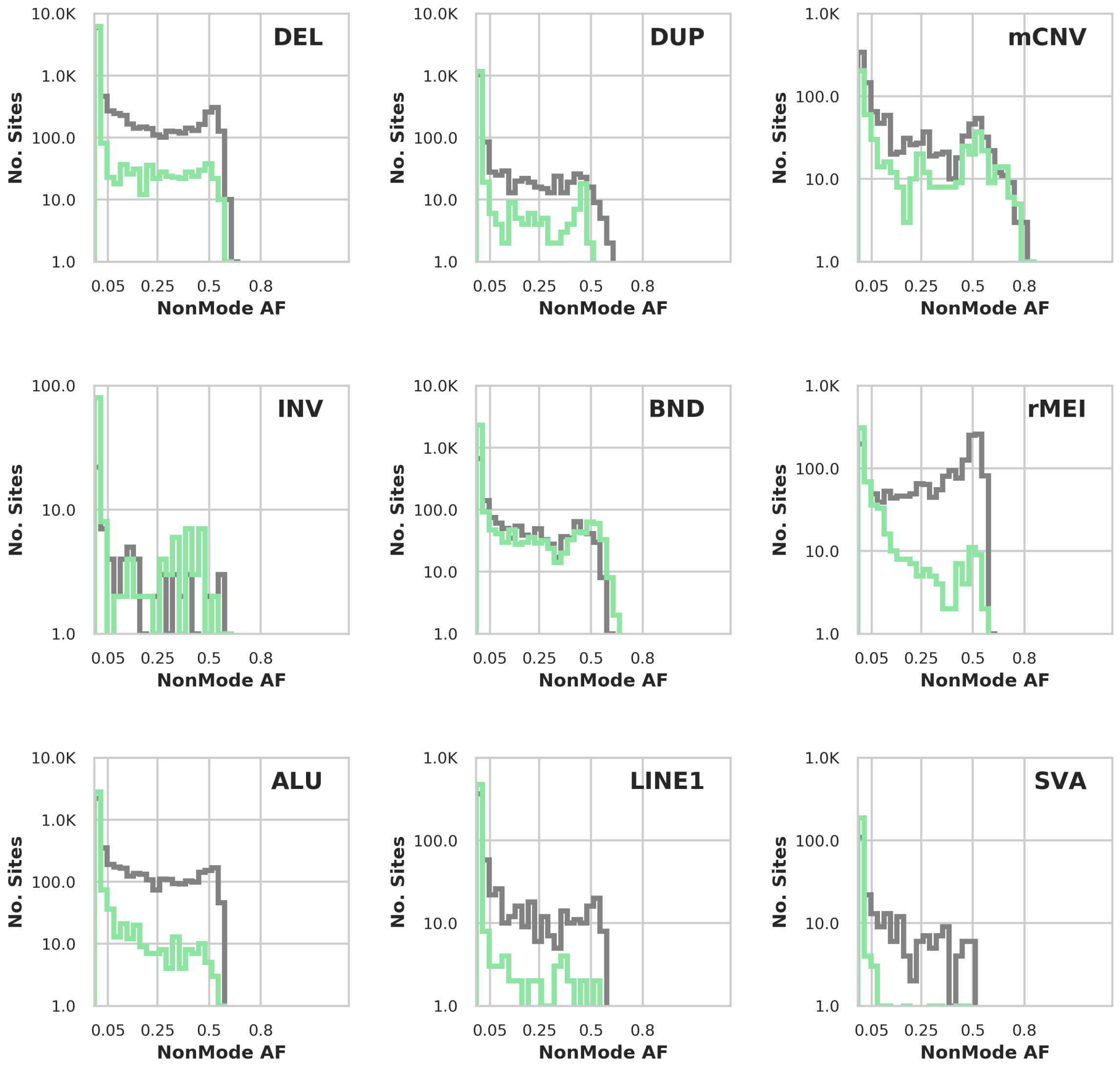

Supplementary Figure 13. Comparing Allele Frequency Distribution of Known and Novel Variants. Non-mode allele frequency distributions of known (defined as overlapping GTEx or 1KGP variants, gray) and novel variants (defined as not overlapping GTEx or 1KGP variants, green) among unrelated i2QTL samples.

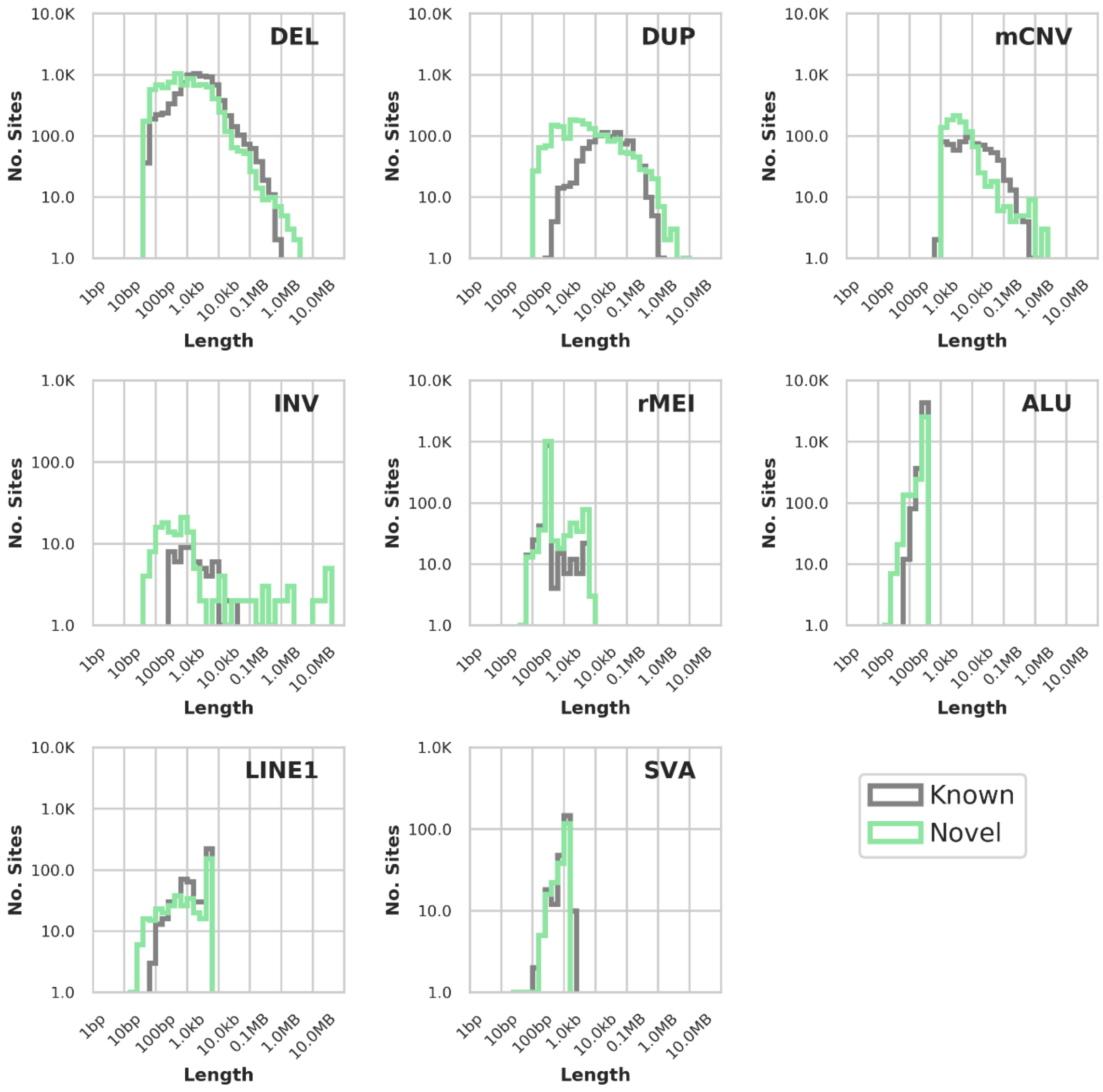

Supplementary Figure 14. Comparing Length Distribution of Known and Novel Variants. Distribution of variant sizes in known and novel variants among unrelated i2QTL samples after intersection with 1000 Genomes Project and GTEx version 6 SV maps.

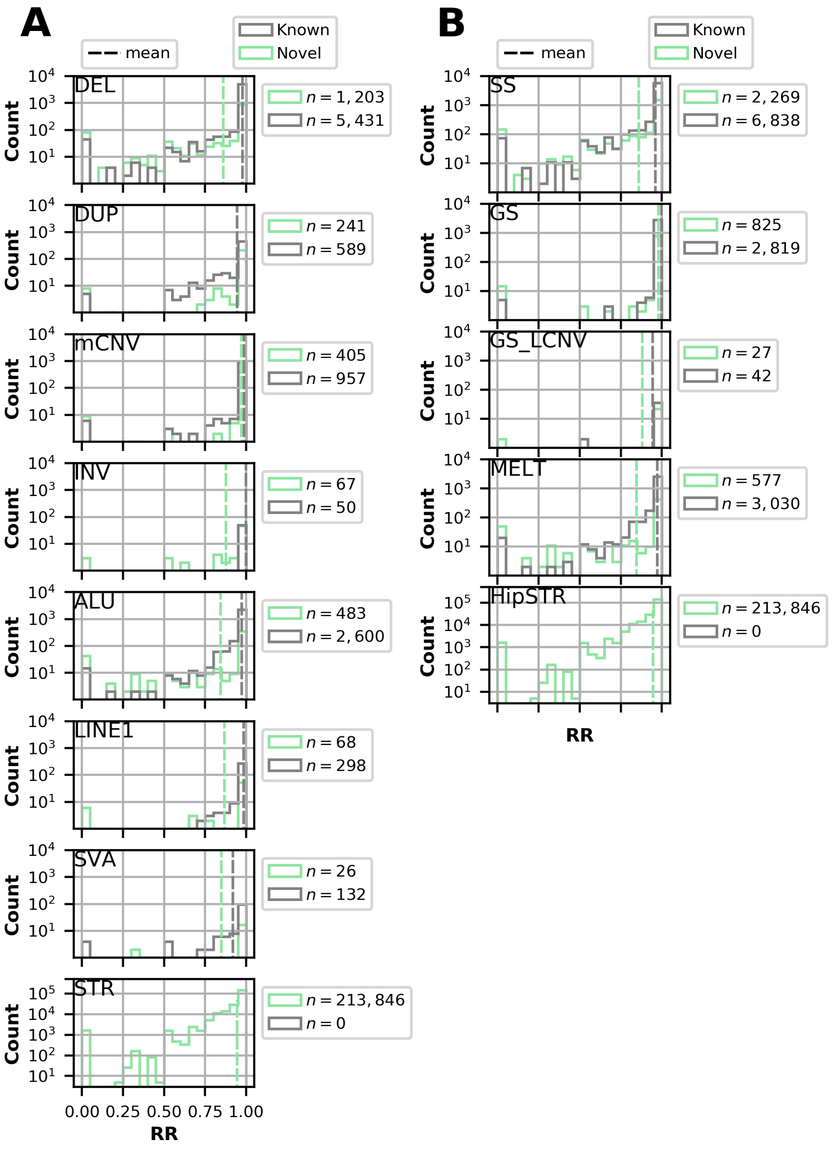

Supplementary Figure 15. Replication Rate of Novel and Known Variants. Distribution or reproducibility rates of known (grey lines) and novel (green lines) variants (A) by caller and (B) by class. The mean reproducibility of each histogram is represented with vertical dashed lines, matching the color of the category. Numbers that are shown in legends indicate the number of variants that segregate in at least one monozygotic twin pair from iPSCORE in each of these categories. Note that only variants segregating in at least one monozgotic twin pair have a replication rate and are thus included in these histograms.

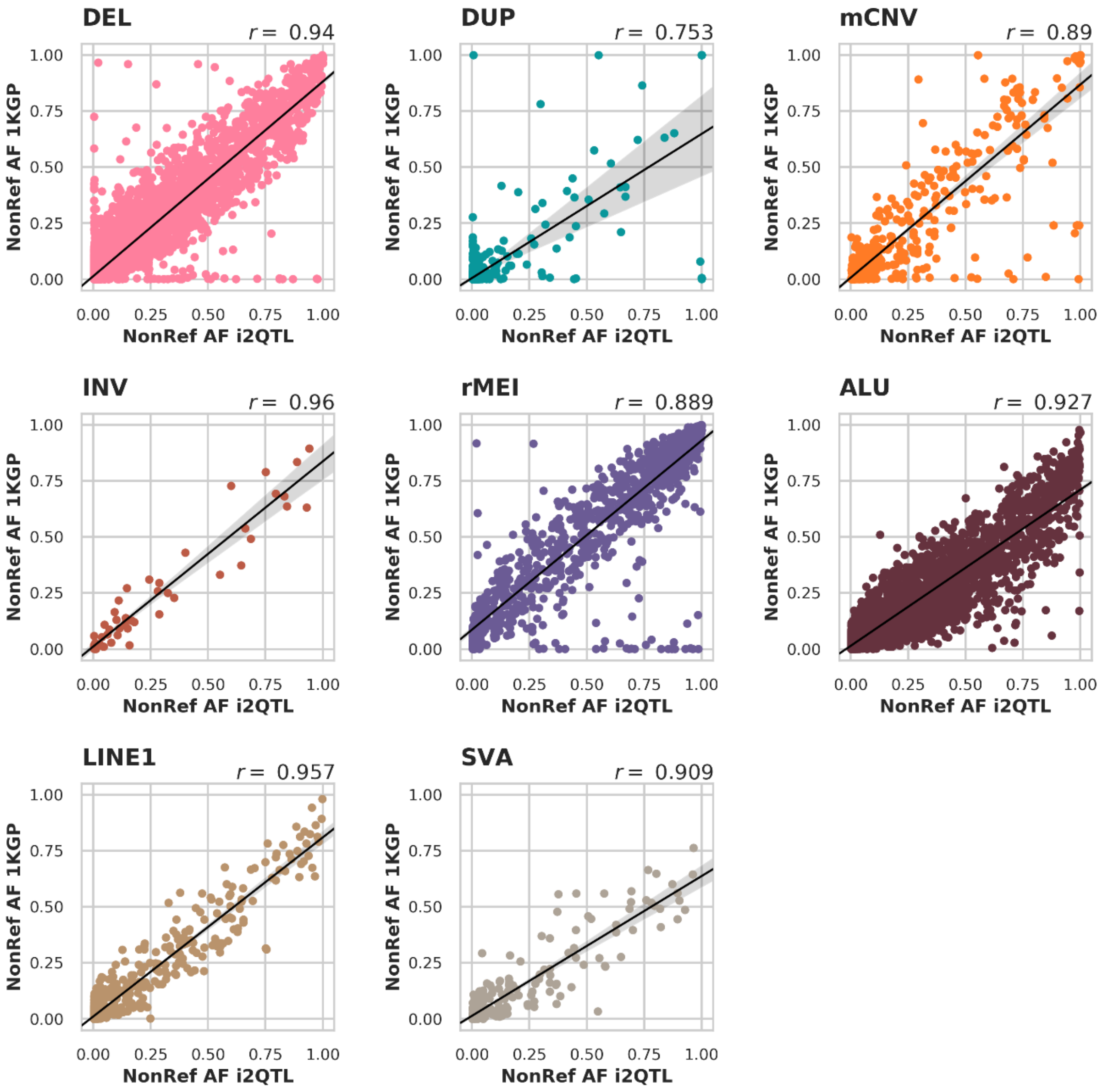

Supplementary Figure 16. Allele Frequency Comparison of Variants Co-Discovered in i2QTL and the 1000 Genomes Project. Non-reference allele frequency of overlapping variants in i2QTL and 1KGP[2], stratified by variant class.

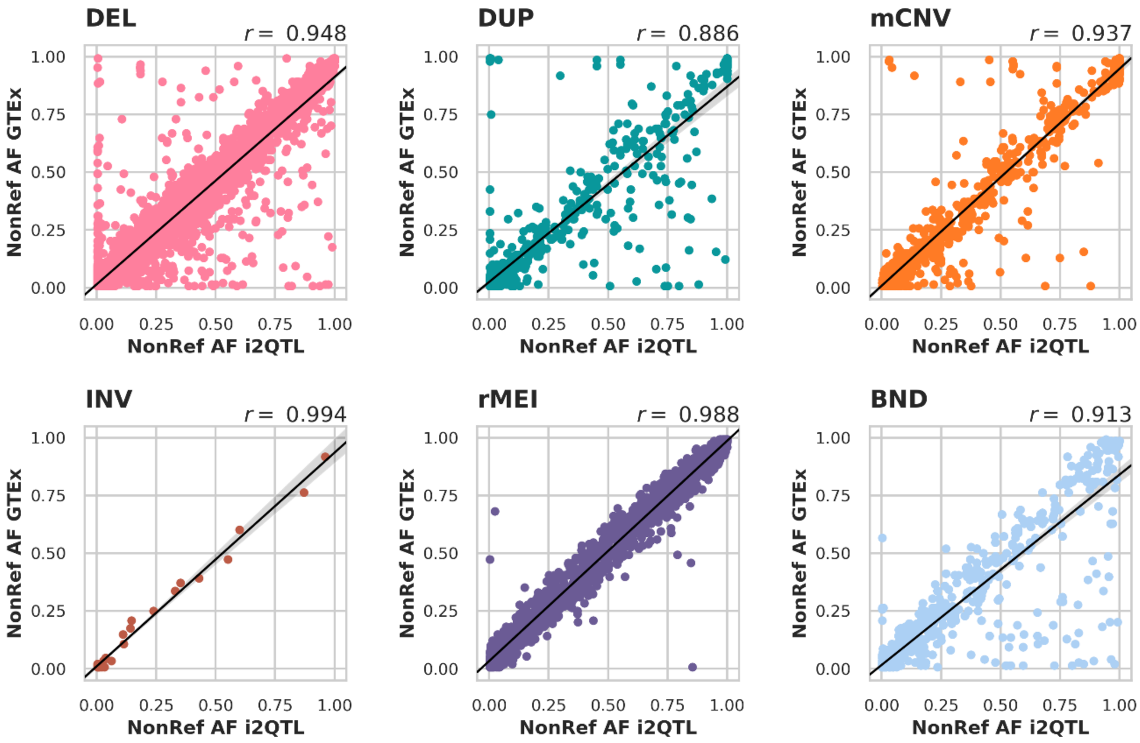

Supplementary Figure 17. Allele Frequency Comparison of Variants Co-Discovered in i2QTL and GTEx. Non-reference allele frequency of overlapping variants in i2QTL and the GTEx V.6 SV call set[3] in their respective cohorts, stratified by variant class. For i2QTL variants, the non-reference allele frequency is computed among unrelated samples.

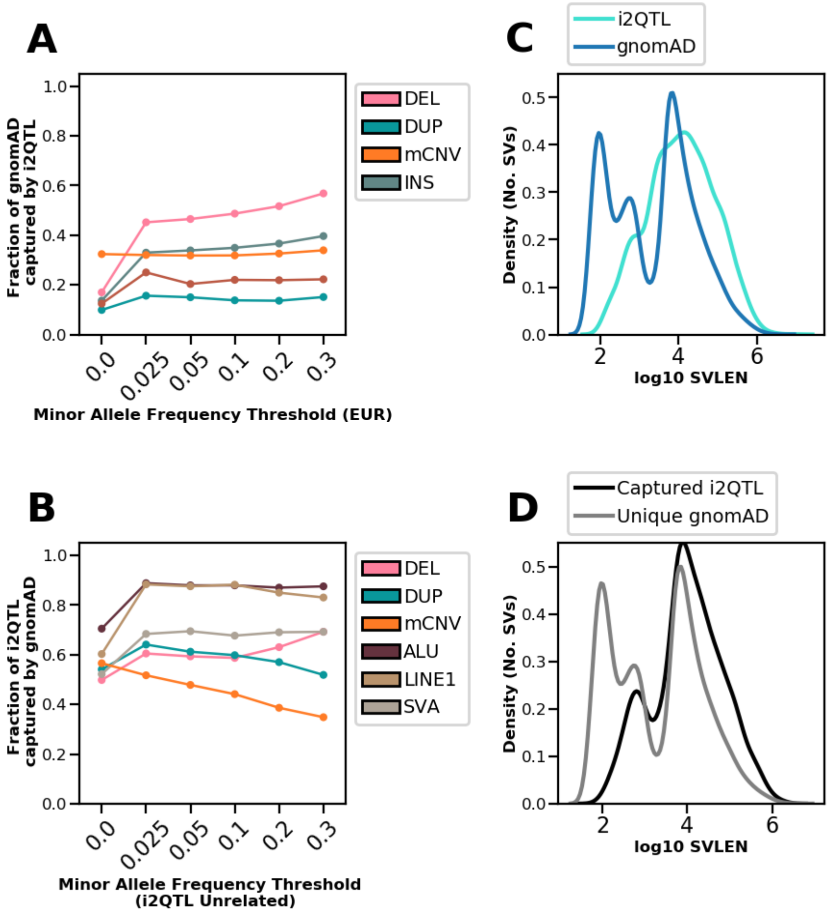

Supplementary Figure 18. Comparison between i2QTL and gnomAD. (A) Proportion of gnomAD SVs of each class co-discovered by i2QTL at different MAF thresholds. (B) Proportion of i2QTL SVs of each class co-discovered by gnomAD at different MAF thresholds. (C) Size distributions of biallelic duplications from gnomAD and i2QTL. (D) Size distribution of biallelic duplications either co-disvovered by i2QTL or unique to gnomAD.

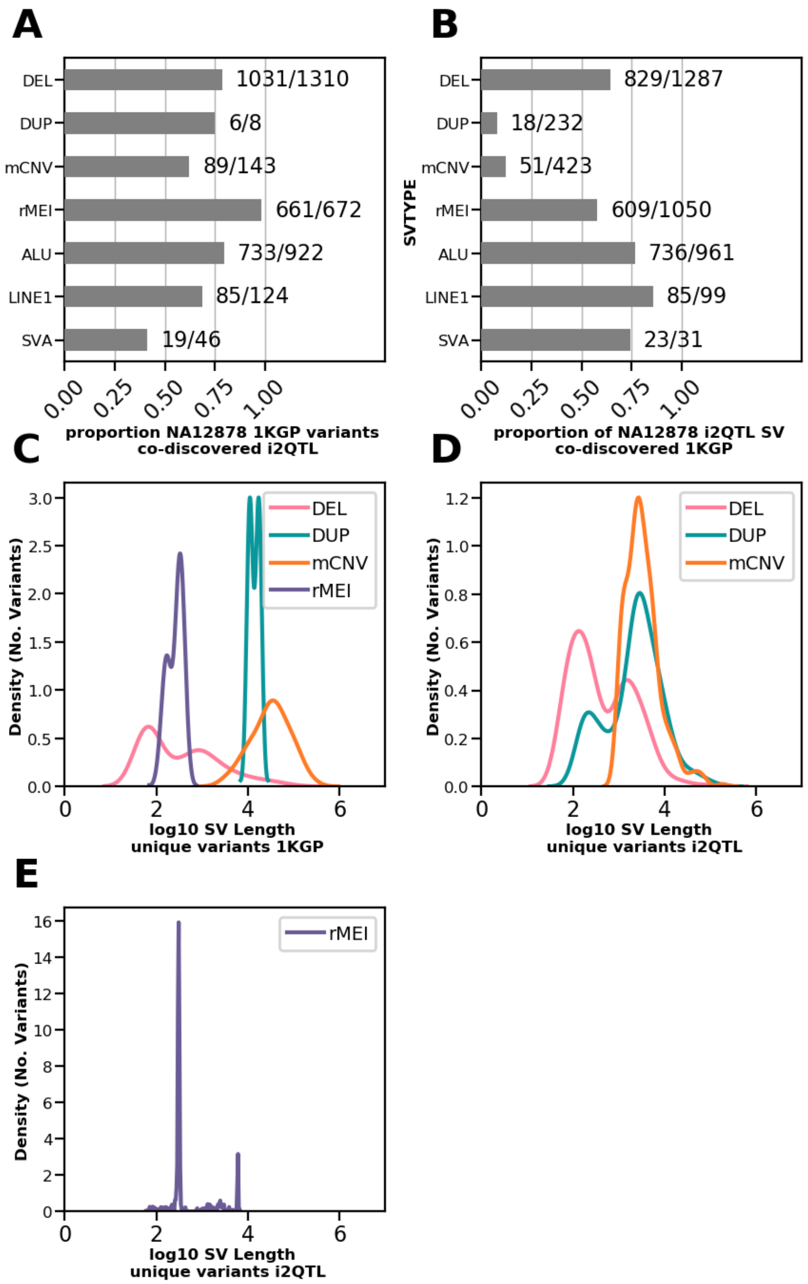

Supplementary Figure 19. Comparison between i2QTL and 1KGP Short-read SVs from NA12878. (A,B) Proportion of SVs of each class from 1KGP co-discovered in i2QTL (A) or proportion of i2QTL SVs co-discovered in 1KGP for sample NA12878 (B). (C,D,E) Size distribution of variants that are unique to 1KGP (C,D) or unique to i2QTL SV datasets (E).

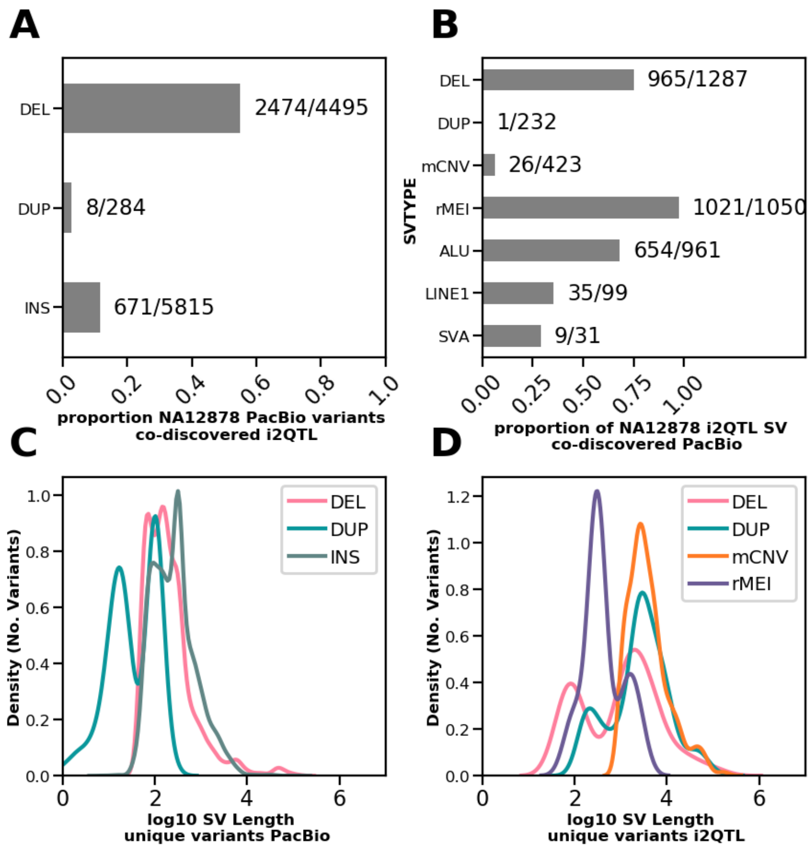

Supplementary Figure 20. Comparison between i2QTL and PacBio Variants for NA12878. (A,B) Proportion of i2QTL SVs of each class co-discovered with PacBio (A) or proportion of PacBio SVs co-discovered in i2QTL for sample NA12878 (B). (C,D) Size distribution of variants that are unique to i2QTL (C) or unique to PacBio SV datasets (D).

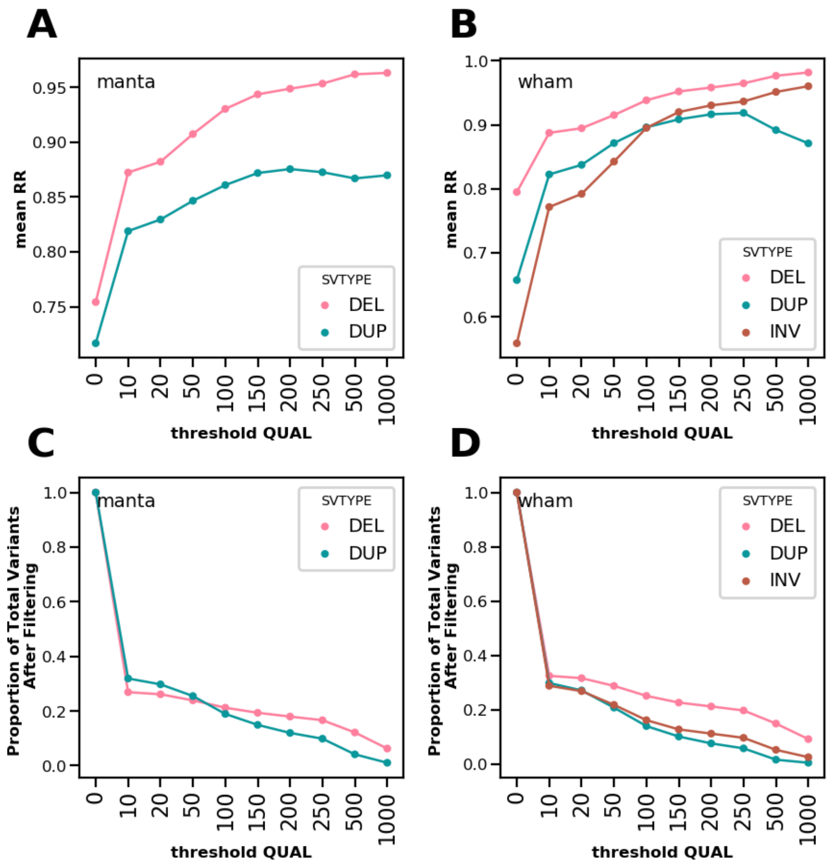

Supplementary Figure 21. Reproducibility of manta and wham Variant Calls. (A,B) Average reproducibility rate (RR) for variants called by manta (A) or wham (B) under different QUAL filtering thresholds for each variant class. (C,D) Proportion of total variants remaining after filtering under each QUAL threshold for manta (C) or wham (D).

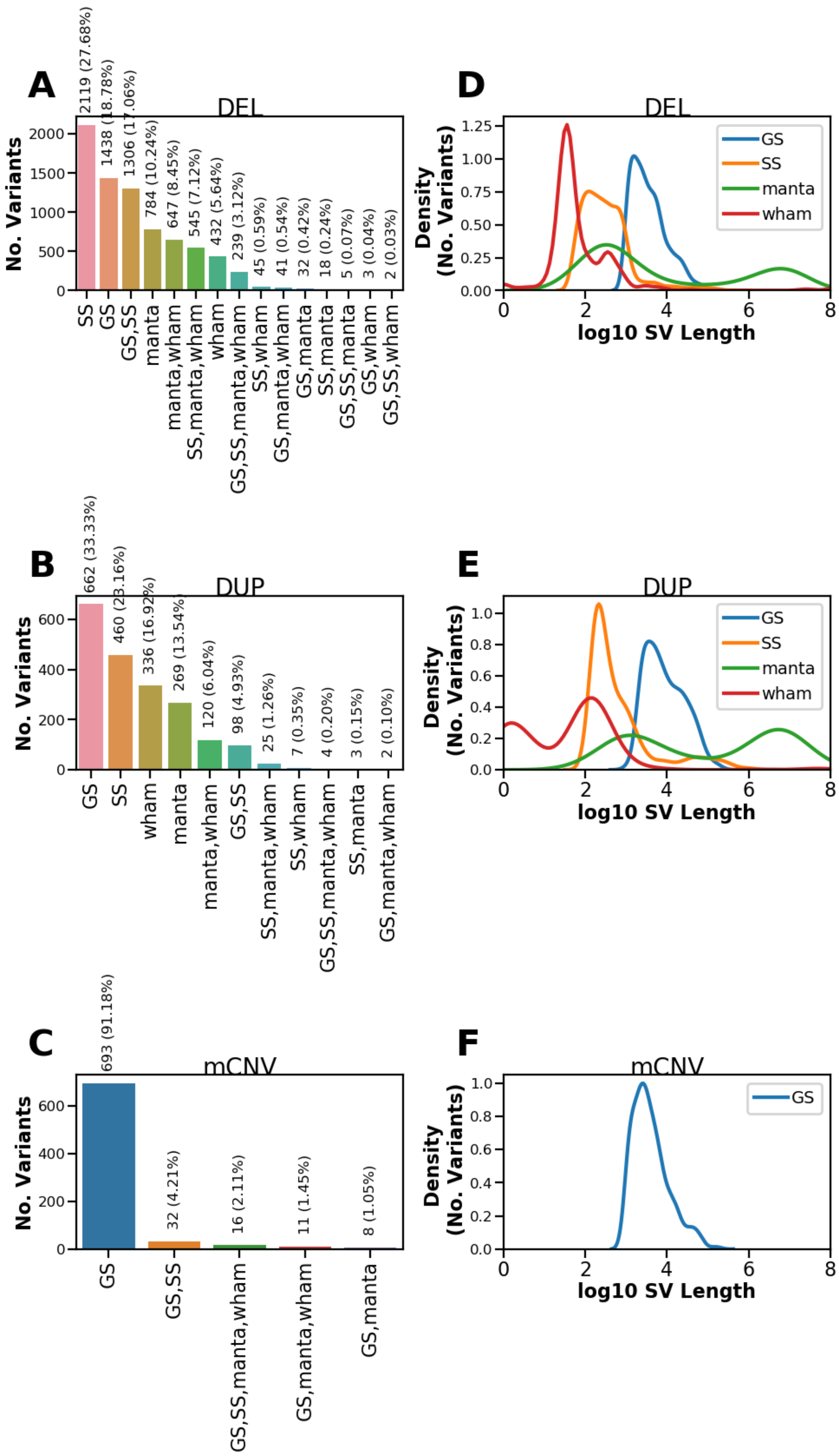
A
DEL
No. Variants
2119 (27.68%)
1438 (18.78%)
1306 (17.06%)
784 (10.24%)
647 (8.45%)
545 (7.12%)
432 (5.64%)
239 (3.12%)
45 (0.59%)
41 (0.54%)
32 (0.42%)
18 (0.24%)
5 (0.07%)
3 (0.04%)
2 (0.03%)
SS
GS
GS,SS
manta
manta,wham
SS,manta,wham
wham
GS,SS,manta,wham
SS,wham
GS,manta,wham
GS,manta
SS,manta
GS,SS,manta
GS,wham
GS,SS,wham
D
DEL
Density (No. Variants)
log10 SV Length
GS
SS
manta
wham
B
DUP
No. Variants
662 (33.33%)
460 (23.16%)
336 (16.92%)
269 (13.54%)
120 (6.04%)
98 (4.93%)
25 (1.26%)
7 (0.35%)
4 (0.20%)
3 (0.15%)
2 (0.10%)
GS
SS
wham
manta
manta,wham
GS,SS
SS,manta,wham
SS,wham
GS,SS,manta,wham
SS,manta
GS,manta,wham
E
DUP
Density (No. Variants)
log10 SV Length
GS
SS
manta
wham
C
mCNV
No. Variants
693 (91.18%)
32 (4.21%)
16 (2.11%)
11 (1.45%)
8 (1.05%)
GS
GS,SS
GS,SS,manta,wham
GS,manta,wham
GS,manta
F
mCNV
Density (No. Variants)
log10 SV Length
GS

Supplementary Figure 22. Overlap between manta, wham, genome STRiP (GS) and SpeedSeq (SS) Calls. (A-C) Number of variants discovered by each algorithm after filtering that have a >0.5 Reciprocal overlap for deletions (A) biallelic duplications (B) and mCNVs (C). (D-F) Distribution of lengths of variants unique to each caller for deletions (D) biallelic duplications (E) and mCNVs (F).

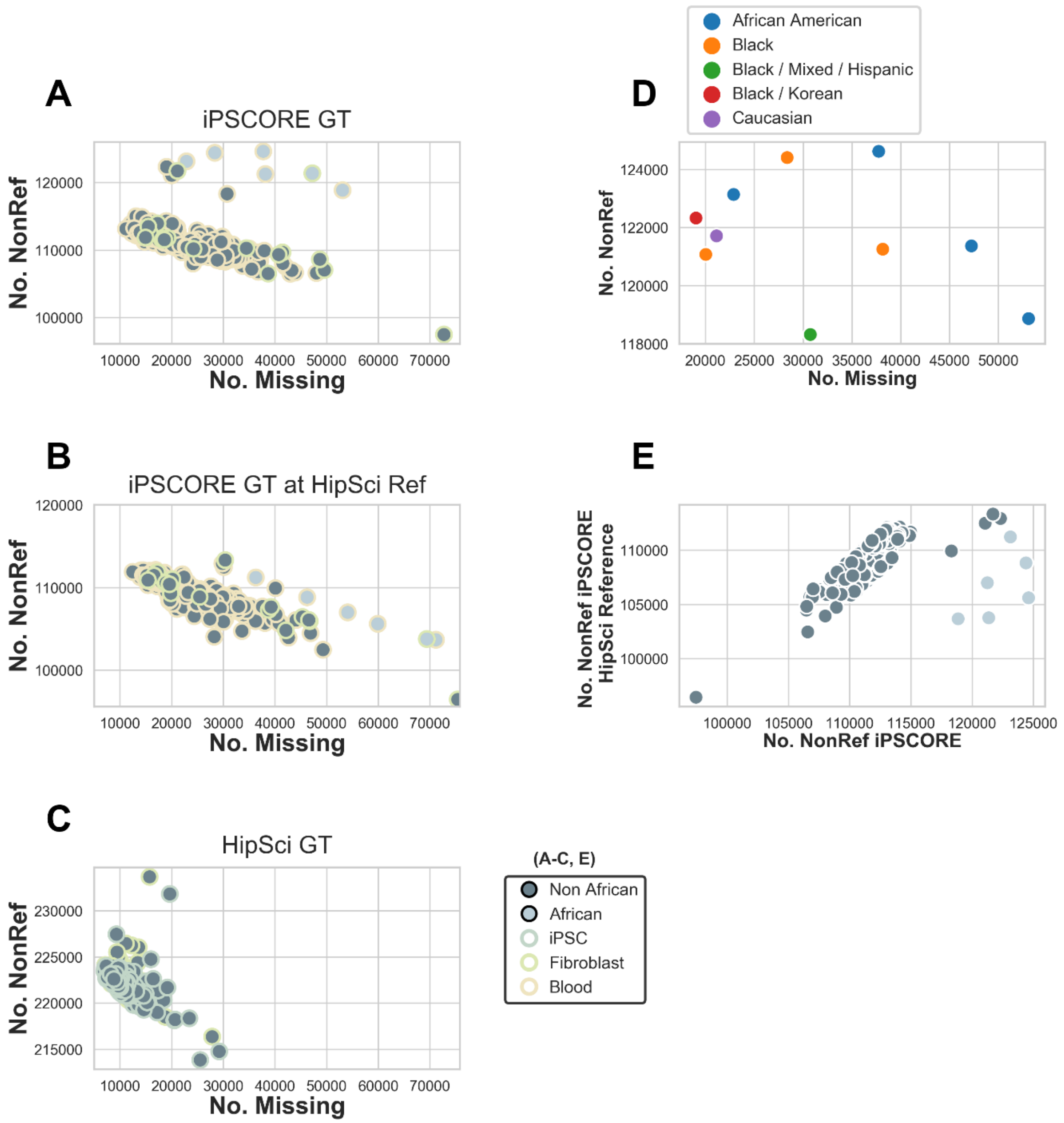

Supplementary Figure 23. HipSTR Quality Control Before Merging Genotypes from iPSCORE/HipSci.(A-C) Number of non-reference calls per sample for HipSTR genotypes on (A) iPSCORE samples, (B) on iPSCORE samples using HipSci genotypes and (C) on HipSci samples. (D) Outliers from A and B are largely samples from individuals with African predicted super population (shown light grey) or that self-reported as partly African. One iPSCORE outlier sample (A, bottom right) was excluded from call rate filtering (80%) of variants. (E) Number of non-reference genotypes discovered in iPSCORE samples versus number discovered in iPSCORE samples by

genotyping HipSci reference alleles. The majority of non-reference sites in iPSCORE were also polymorphic in the HipSci sample set, and the genotypes were similar, however, variants unique to the African samples are not well represented (shown in light grey), as none of the HipSci samples were of African ancestry.

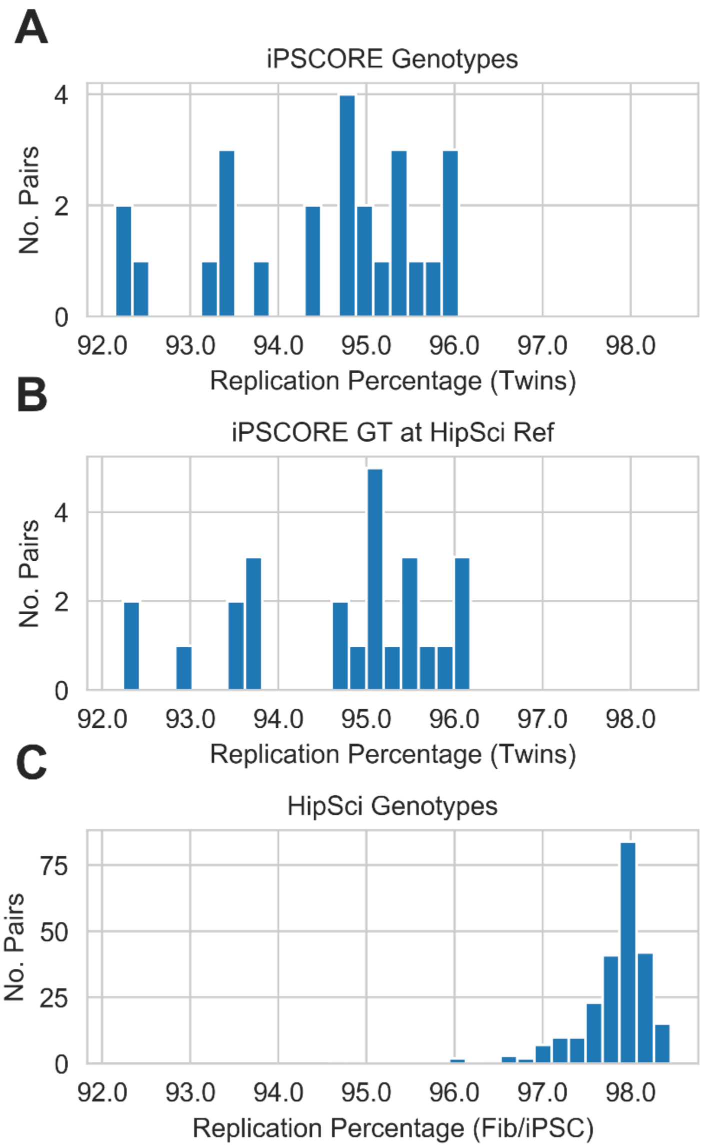

Supplementary Figure 24. HipSTR Replication Rates in Twin Pairs and iPSC Fibroblast Pairs for Different Genotyping Subsets. (A-C) Replication rate per twin

pair after quality filtering for HipSTR genotypes (A) on iPSCORE samples, (B) on iPSCORE samples using HipSci genotypes and (C) on HipSci samples. Here, we observed higher replication percentages among HipSci samples, due to the PCR free protocol of these WGS samples.

Supplementary Tables

Variant Caller	Variant Class	Applied	Filtering Parameter	Threshold	Rationale
Genome STRiP CNVDiscovery	DEL	Per Site	GSCNQUAL	2	RR
	DUP	Per Site	GSCNQUAL	14	RR
	mCNV	Per Site	GSCNQUAL	12	RR
	ALL	Per Site	% Low Quality Genotypes (LQ tag)	< 10%	
Genome STRiP LCNVDiscovery	ALL	Per Site	Contained in Centromere	N/A	Suggested (Methods)
	ALL	Per Site	NBINS	≥ 10	Suggested (Methods)
	ALL	Per Site	SCORE	≥ 1000	Suggested (Methods)
	ALL	Per Genotype	Absolute Copy Number	1.25 < or > 2.75	Suggested (Methods)
SpeedSeq	DEL	Per Site	MSQ	20	RR
	DUP	Per Site	MSQ	100	RR
	rMEI	Per Site	MSQ	20	RR
	INV	Per Site	MSQ	90	RR
	BND	Per Site	MSQ	90	RR
	BND	Per Site	≥ 25% PE/SR support	N/A	Chiang et al. 2017
	INV	Per Site	QUAL	>100	Chiang et al. 2017
	INV	Per Site	≥ 10% PE or SR support	N/A	Chiang et al. 2017
	ALL	Per Site	% Missing	< 10%	
MELT	ALL	Per Site	low complexity (lc)	N/A	RR/Gardner et al. 2017
	ALL	Per Site	>25% missing (s25)	N/A	RR/Gardner et al. 2017
	ALL	Per Site	LP/ RP > 2.0 standard deviations (rSD)	N/A	RR/Gardner et al. 2017
	ALL	Per Site	more discordant pairs are also split than expected (hDP)	N/A	RR/Gardner et al. 2017
	ALL	Per Site	ASSESS	=5	RR
HipSTR	ALL	Per Genotype	call quality (Q)	0.9	RR/Willems et al. 2017
	ALL	Per Genotype	Fraction reads with flanking indel (DFLANKINDEL/DP)	≤ 0.15	
	ALL	Per Genotype	Fraction Reads With Stutter (DSTUTTER/DP)	≤ 0.15	
	ALL	Per Genotype	log10 allele bias p-value (AB)	>-2	
	ALL	Per Genotype	log10 strand bias p-value (FS)	>-2	

Supplementary Table 1. **Filtering parameters overview**. Table describing the filtering parameters used when generating the i2QTL SV/STR call set, stratified by variant caller and variant class when necessary. The "Applied" column indicates whether the filter was applied to set specific low-quality genotypes to missing on a per sample basis within each variant class ("Per Genotype" filters) or whether the filter was used to filter an entire site for removal from downstream analysis ("Per Site" filtering). The column "Filtering Parameter" indicates the specific filter.

Supplementary References

1 Auton, A. *et al.* A global reference for human genetic variation. *Nature* **526**, 68-74, doi:10.1038/nature15393 (2015).

2 Sudmant, P. H. *et al.* An integrated map of structural variation in 2,504 human genomes. *Nature* **526**, 75-81, doi:10.1038/nature15394 (2015).

3 Chiang, C. *et al.* The impact of structural variation on human gene expression. *Nature Genetics* **49**, 692-699, doi:10.1038/ng.3834 (2017).
