# Supplementary material for: Discovery and quality analysis of a comprehensive set of structural variants and short tandem repeats
Source: Nat Commun. 2020 Jun 10;11:2928. doi: 10.1038/s41467-020-16481-5 (PMC7287045; doi:10.1038/s41467-020-16481-5)
Supplement: Supplementary file 2 — Description of Additional Supplementary Information [file 41467_2020_16481_MOESM2_ESM.pdf]

Description of Additional Supplementary Files

File Name: Supplementary Data 1
Description: Subject Information. Phenotypic information about 477 individuals from iPSCORE and HipSci with WGS used in this study including age, sex, predicted superpopulation, reported and annotation of family, twin status, presence in the unrelated set.

File Name: Supplementary Data 2
Description: Whole Genome Sequencing Information. Information describing WGS samples included in variant calling including cell type, subject, study, and also median coverage for each genome.

File Name: Supplementary Data 3
Description: Non-redundant Structural Variants. Table describing the non-redundant variants including position, variant class, variant caller, and evidence supporting the site.
