# Supplementary material for: Discovery and quality analysis of a comprehensive set of structural variants and short tandem repeats
Source: Nat Commun. 2020 Jun 10;11:2928. doi: 10.1038/s41467-020-16481-5 (PMC7287045; doi:10.1038/s41467-020-16481-5)
Supplement: Supplementary file 6 — Reporting Summary [file 41467_2020_16481_MOESM6_ESM.pdf]

nature research

Corresponding author(s): Kelly A. Frazer

Last updated by author(s): Aug 6, 2019

Reporting Summary

Nature Research wishes to improve the reproducibility of the work that we publish. This form provides structure for consistency and transparency in reporting. For further information on Nature Research policies, see Authors & Referees and the Editorial Policy Checklist .

Statistics

For all statistical analyses, confirm that the following items are present in the figure legend, table legend, main text, or Methods section.

n/a	Confirmed	
x		The exact sample size (*n*) for each experimental group/condition, given as a discrete number and unit of measurement
x		A statement on whether measurements were taken from distinct samples or whether the same sample was measured repeatedly
	x	The statistical test(s) used AND whether they are one- or two-sided<br>*Only common tests should be described solely by name; describe more complex techniques in the Methods section.*
	x	A description of all covariates tested
	x	A description of any assumptions or corrections, such as tests of normality and adjustment for multiple comparisons
	x	A full description of the statistical parameters including central tendency (e.g. means) or other basic estimates (e.g. regression coefficient) AND variation (e.g. standard deviation) or associated estimates of uncertainty (e.g. confidence intervals)
	x	For null hypothesis testing, the test statistic (e.g. *F*, *t*, *r*) with confidence intervals, effect sizes, degrees of freedom and *P* value noted<br>*Give P values as exact values whenever suitable.*
x		For Bayesian analysis, information on the choice of priors and Markov chain Monte Carlo settings
x		For hierarchical and complex designs, identification of the appropriate level for tests and full reporting of outcomes
	x	Estimates of effect sizes (e.g. Cohen's *d*, Pearson's *r*), indicating how they were calculated

Our web collection on statistics for biologists contains articles on many of the points above.

Software and code

Policy information about availability of computer code

Data collection	No software was used for data collection.
Data analysis	sambamba(v0.5.9); biobambam2 (v2.0.21); HipSTR(v0.6.1); GATK; Genome STRiP(svtoolkit 2.00.1611); MELT (v2.0.2); SpeedSeq(v0.1.2); LUMPY(v0.2.13); svtools(v0.3.2, https://github.com/hall-lab/svtools/tree/v0.3.2, https://doi.org/10.5281/zenodo.376864), CNVnator(v0.3.3);GenomeStudio (v2011.1); GitHub:https://github.com/frazer-lab/i2QTL-SV-STR-analysis<br>Python(v2.7.15); Python Packages: seaborn (v0.9.0, https://pypi.org/project/seaborn/0.9.0); statsmodels (v0.9.0, https://pypi.org/project/statsmodels/0.9.0); scipy(v1..1.0, https://www.scipy.org); matplotlib(v2.2.3, https://pypi.org/project/matplotlib/2.2.3); pandas (v0.22.0, https://pypi.org/project/pandas/0.22.0

For manuscripts utilizing custom algorithms or software that are central to the research but not yet described in published literature, software must be made available to editors/reviewers. We strongly encourage code deposition in a community repository (e.g. GitHub). See the Nature Research guidelines for submitting code & software for further information.

Data

Policy information about availability of data

All manuscripts must include a data availability statement. This statement should provide the following information, where applicable:
- Accession codes, unique identifiers, or web links for publicly available datasets
- A list of figures that have associated raw data
- A description of any restrictions on data availability

Structural variant calls are available at dbGaP accession phs001325.

Field-specific reporting

Please select the one below that is the best fit for your research. If you are not sure, read the appropriate sections before making your selection.

- [x] Life sciences
- [ ] Behavioural & social sciences
- [ ] Ecological, evolutionary & environmental sciences

For a reference copy of the document with all sections, see nature.com/documents/nr-reporting-summary-flat.pdf

Life sciences study design

All studies must disclose on these points even when the disclosure is negative.

Sample size	We used all available samples from the IPSCORE and HipSci datasets with whole genome sequencing data.
Data exclusions	We excluded one iPSCORE sample from the STR missingness calculation that had more than 70,000 missing calls for STRs.
Replication	Structural variant call replication was assessed using replication rate as described in the manuscript. The replication rate is calculated as the proportion of non-reference genotypes that were also called non-reference in a paired genetic replicate.
Randomization	Samples were randomized during sample processing but otherwise randomization was not applicable in this study. As described in the manuscript, we assessed whether batch effects were present between the iPSCORE and HipSci datasets.
Blinding	Blinding was not performed and was not relevant in this study because we were not studying outcomes.

Reporting for specific materials, systems and methods

We require information from authors about some types of materials, experimental systems and methods used in many studies. Here, indicate whether each material, system or method listed is relevant to your study. If you are not sure if a list item applies to your research, read the appropriate section before selecting a response.

Materials & experimental systems

n/a	Involved in the study	
x		Antibodies
x		Eukaryotic cell lines
x		Palaeontology
x		Animals and other organisms
	x	Human research participants
x		Clinical data

Methods

n/a	Involved in the study	
x		ChIP-seq
x		Flow cytometry
x		MRI-based neuroimaging

Human research participants

Policy information about studies involving human research participants

Population characteristics	In total, we utilized a total of 477 HipSci and iPSCORE subjects, 276 were females and 201 were males, and collectively subjects ranged in age from 5 and 89 years of age (Figure S1A). Notably, iPSCORE individuals were included in 56 families composed of two or more subjects (range: 2 to 14 subjects) and 86 single individuals (Figure S1B, Table S1). Overall, 167 iPSCORE individuals were unrelated. All iPSCORE individuals were grouped into one of five superpopulations (European, African, Admixed American, East Asian, and South Asian) on the basis of genotype data (D'Antonio et al., 2018; DeBoever et al., 2017; Panopoulos et al., 2017b) and HipSci samples were similarly categorized (Kilpinen et al., 2017a) (Figure S1C). For HipSci, some subjects had multiple iPSC clones with WGS. For these subjects, we chose the pair of fibroblast and iPSC WGS samples that had the highest reproducibility for Genome STRiP calls (see Section 3.3 below).
Recruitment	273 subjects were recruited as part of the iPSCORE study, of which 215 subjects have been previously described (D'Antonio et al., 2018; DeBoever et al., 2017; Panopoulos et al., 2017b). Data for additional 204 subjects was obtained from the HipSci Collection (Kilpinen et al., 2017a; Streeter et al., 2017).
Ethics oversight	The iPSCORE collection was approved by the Institutional Review Board of the University of California at San Diego (Project #110776ZF).

Note that full information on the approval of the study protocol must also be provided in the manuscript.
